# How Does Landscape Structure Affect Dung Beetle Assemblages in Amazon Cities?

**DOI:** 10.1002/ece3.70704

**Published:** 2025-01-08

**Authors:** Vanessa Pontes Mesquita, Glenda Vanessa dos Santos Bernardino, Paulo Estefano Dineli Bobrowiec, Renato Portela Salomão, Cintia Cornelius

**Affiliations:** ^1^ Programa de Pós‐Graduação em Ecologia Instituto Nacional de Pesquisas da Amazônia Manaus Brazil; ^2^ Facultad de Estudios Superiores Iztacala Universidad Nacional Autónoma de México Tlalnepantla de Baz Mexico; ^3^ Instituto de Ciências Biológicas Universidade Federal do Amazonas Manaus Brazil

**Keywords:** biotic homogenization, community ecology, rainforest, Scarabaeinae, urban sprawl

## Abstract

The growth of cities is one of the main direct and indirect factors responsible for the loss of native vegetation cover. Urbanization directly affects the biological communities inhabiting forest remnants inserted in cities, compromising the maintenance of urban and natural ecosystems. By understanding the effects of landscape transformation due to urbanization, we can have insights regarding the distribution of land uses that allow a proper maintenance of the urban ecosystems. This work assessed the effects of landscape structure variables (forest cover, agricultural area, edge density, and number of forest patches) on dung beetle assemblages and functional groups (i.e., diet and resource removal strategy) sampled in 38 sites located along an urban–rural gradient of six cities belonging to the metropolitan area of Manaus in Central Amazonia. Losses of forest cover were the most determining factor, negatively affecting species richness, abundance, and body size. The increases in agriculture cover negatively affected dung beetle abundance, while edge density positively affected their abundance. The number of forest patches positively affected dung beetle abundances—except for dweller species—and negatively affected the body size of diet‐generalist species. These results demonstrate that changes in ecological diversity caused by urbanization are driven mostly by forest cover loss, although forest configuration is important for dung beetle abundance. This study contributes to the understanding of how changes in the amount and distribution of forest cover in tropical cities affect the taxonomic diversity of dung beetle assemblages.

## Graphical abstract

The increase of urbanization in Amazonian cities present drastical decreases in dung beetle diversity. Forest cover is the main landscape metric that drives dung beetle diversity in Amazonian cities.

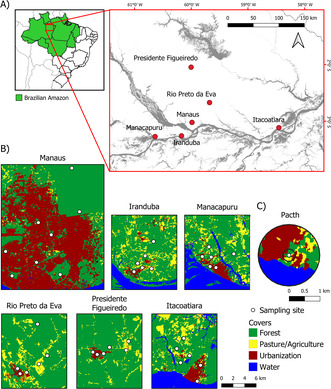

## 1 Introduction

Land cover changes caused by the advance of cities are one of the main factors related to urbanization that impacts biodiversity (Müller et al. 2018). The accelerated growth of cities is linked to forest loss and fragmentation, generating forest remnants surrounded by a matrix composed mostly of impervious surfaces (Parris Kirsten 2016; Magura, Ferrante, and Lövei 2020), incapable of being used by communities that dwell in forested environments. Through different land uses, human activities alter processes and the stability of natural ecosystems (Deng et al. 2021). Consequently, there is a loss or reduction of suitable habitat for native communities, introduction of exotic species, and changes in habitat quality and food availability (McKinney 2002; Kowarik 2011; Rocca and Milanesi 2022). These processes observed in urban environments tend to negatively affect species richness and abundance of ecological communities (Violle et al. 2007; McKinney 2008; Aronson et al. 2014).

In the Brazilian Amazon, urbanization evolved with the emergence of intermediate‐sized cities and the multiplication of small urban centers (Sathler, Monte‐Mór, and Carvalho 2009). Despite being a region that has historically been altered by human activities (Macedo and Filippi 2021), the Amazon still has a large portion of native forest cover. The nonurbanized regions around the Amazonian cities have a relatively pristine condition, especially when compared to other tropical forest regions (Vitel, Fearnside, and Graça 2009; Levis et al. 2017). Thus, the forest matrix that surrounds the cities of this ecosystem represents a unique scenario regarding the dynamics of urbanization in the tropics. Through knowledge about the effects of forest loss in Amazonian cities, it is possible to deepen the understanding of the resilience of ecological communities in the face of abrupt changes in tropical landscapes.

Landscape ecology stands out as an optimal approach that helps to understand how the spatial organization of landscape units influences species distribution (Metzger 2001). For example, in unmanaged forest areas, the impact of urbanization is different compared with intensively managed agricultural land (Venugopal, Thomas, and Flemming 2012). With the increase in cities, original forest cover is converted into urban settlements and surrounding agricultural landscapes, generating smaller native remnants isolated from each other by an urban matrix (Laurance, Vasconcelos, and Lovejoy 2000; Laurance, Goosem, and Laurance 2009). Together with such changes, there is a loss of forest cover and an increase in the density of edges (i.e., the length of edges in a landscape) surrounding the forest remnants embedded in cities, impacting the structure and composition of forests and its communities (Harper et al. 2005). Such changes in biodiversity directly imply the provision of ecological services (e.g., food and raw material production, climate regulation, soil conservation, and biodiversity) provided by native communities (Tolessa, Senbeta, and Kidane 2017; Liu et al. 2019). By assessing the effects of landscape transformation due to urbanization, we can have cues regarding the best distribution of land uses that will allow the proper maintenance of the urban ecosystems.

Among invertebrates, the dung beetles (Coleoptera: Scarabaeinae) are considered excellent bioindicators of man‐made environmental changes (Gardner et al. 2008; Viegas et al. 2014; Noriega et al. 2020). This group is classified into functional groups according to their diet (i.e., coprophagous, scavenger, and generalist) and resource removal strategy (i.e., telecoprids, paracoprids, and endocoprids) (Santos‐Heredia et al. 2018; Carvalho et al. 2020). The telecoprids (rollers) manipulate small portions of the resource, which are formed into spheres and rolled away from the sources until they are buried in the ground; paracoprids (tunnelers) dig underground tunnels just below the source; and endocoprids (dwellers) feed and nest directly in the fecal mass (Halffter and Edmonds 1982; Batilani‐Filho 2015). Through the use of mammalian feces by dung beetles, a number of ecological functions are performed by these insects, such as nutrient cycling, parasite suppression, and soil aeration (Hanski and Cambefort 1991; Nichols et al. 2009). Moreover, the use of functional groups has proven to be an important tool for understanding the effects of environmental disturbances on biological communities (Podgaiski, De Souza Mendonça Jr, and De Patta Pillar 2011), allowing a finer understanding of ecological dynamics compared to assemblage structure as a whole (Vaz‐de‐Mello et al. 2009).

The landscape effects associated with deforestation activities (e.g., increase in edge density and nonforested matrices, and decrease in forest cover) can lead to a reduction in dung beetle biomass and diversity (Sánchez‐de‐Jesús et al. 2016; Silva et al. 2019; Souza et al. 2020). Although previous studies have shown negative effects of urbanization on dung beetles in tropical ecosystems (Korasaki et al. 2013; Salomão et al. 2019; Correa, Silva, et al. 2021), to our knowledge no studies analyzed these processes and patterns in Amazonian landscapes. The goal of this study was to evaluate the effects of landscape composition and configuration on the dung beetle assemblages that inhabit different forest areas along an urban–rural gradient in an urbanizing Amazonian landscape. We tested how the amount of forest cover, agricultural cover, edge density, and the number of forest patches affect dung beetle assemblage structure, species richness, abundance, species composition, and body size. Landscape effects on dung beetles were analyzed using both the entire assemblage and each functional group, according to beetles' resource removal strategy and diet. We expect that the decrease in forest cover in Amazonian urban forest fragments will decrease dung beetle species richness, abundance, and body size.

## 2 Materials and Methods

### 2.1 Study Area

**FIGURE 1:**
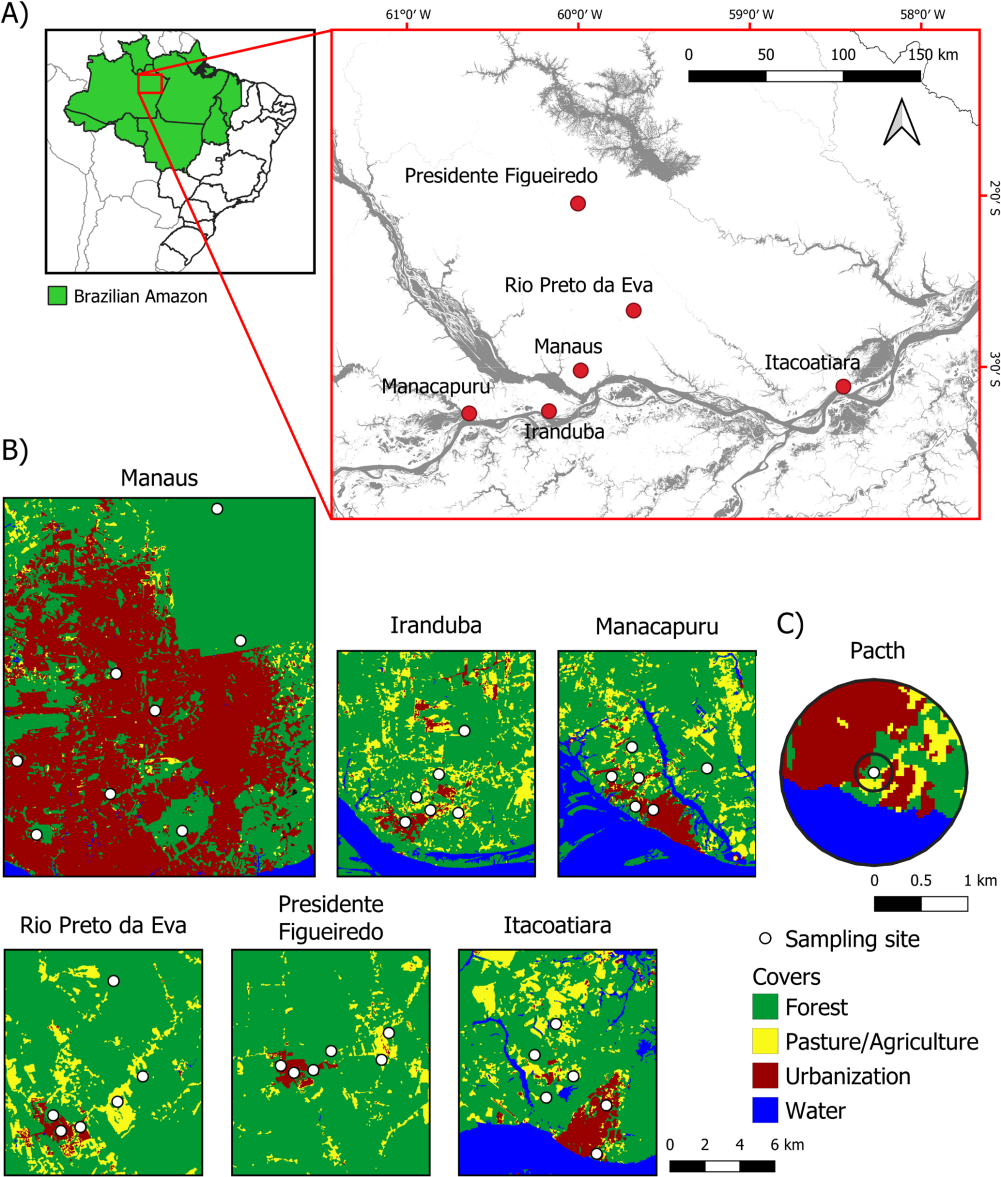
Map showing the location of the visited cities (red points) in the state of Amazon, Brazil (A); each white point represents a study site. Detailed map of the six Amazonian cities where the study was carried out (B), and example of landscape in which dung beetles were sampled (C). Figure obtained from Bernardino (2024).

The study was conducted in the state of Amazon, Brazil, located in the urban and periurban areas of the cities of Iranduba, Itacoatiara, Manacapuru, Manaus, Presidente Figueiredo, and Rio Preto da Eva (Figure 1). City areas range from 2216 to 25,459 km^2^ (IBGE 2022), and the number of inhabitants ranges from 34,856 to 2,255,903 (IBGE 2022). According to the classification proposed by Köppen, the climate is humid tropical (Am), with an average annual rainfall of 2286 mm and temperature varying between 27°C and 29°C (INMET 2011). The rainy season in the region occurs between November and March (mean monthly rainfall: 299 mm), and the dry period occurs between May and September (mean monthly rainfall: 93 mm). Forest type studied comprises mostly *terra firme* forest. The matrix in our studied landscapes is composed mainly of urban cover, agricultural lands, and water cover. Sampling sites were placed in forest areas along a gradient of urbanization (from areas with lower forest amounts—more urbanized, towards areas with higher forest amounts—less urbanized). Sampling sites were located from the central portions of the cities (with city being the matrix) to the city surroundings (conserved forest being the matrix).

Despite intense changes in land use due to the continuous process of urbanization in the studied landscapes, urban surroundings are mostly dominated by native vegetation. Land cover not only comprises mostly primary and secondary forests but also contains agricultural cover (Figure 1). Forest fragments in this landscape are sometimes small vegetation remnants, but they maintain similar structural complexity to tropical forests. Trees, palms, and herbs form different forest strata from the understory to the canopy, and the density and diversity of species vary across space, creating complex ecosystems (Oliveira‐Filho et al. 2021; Costa, Colares, and Monteiro 2022).

### 2.2 Data Collection

We surveyed dung beetles between March and June 2021, at the end of the rainy period. The dung beetles of tropical rainforests are active in this period, thus being appropriated to obtain a representative sample of their assemblages (Ratcliffe 2013; Iannuzzi et al. 2016). We sampled beetles in 38 sites (six sites per city, except for Manaus, in which we sampled eight sites, see Figure 1). Sites were always located within forested areas at a minimum distance of 20 m from forest edges and were sampled once during the entire study. To capture the dung beetles, we used pitfall traps. Traps consisted of a cylindrical plastic container (500 mL, 11 cm diameter × 11 cm height) buried at the ground level in the soil, with a small bait‐holding recipient installed. Inside the cylindrical plastic container, we used 250 mL of a solution containing salt and detergent to kill and preserve the collected beetles. To attract the dung beetles, traps were baited with ca. 25 g of human excrement or ca. 25 g of carrion (decaying bovine liver). In each sampling site, 10 paired traps baited with excrement and carrion (in each pair, traps spaced 10 m between them) were installed each 25 m across a 250‐m‐long linear transect. The beetles that fell in traps were collected 48 h after the installation of the pitfall traps. A total of 760 traps (20 traps × 38 fragments) were installed in this study.

### 2.3 Functional Groups and Estimation of Species' Body Size

We identified beetles at species level with the aid of taxonomic keys (Génier 2009; Edmonds and Zidek 2010; Vaz‐de‐Mello et al. 2011; Cupello and Vaz‐de‐Mello 2019; Nazar‐Silva and Silva 2021), taxonomic experts (Dr. Mario Cupello, Texas A&M University), and reference material of the Entomological Collection of the Instituto Nacional de Pesquisas da Amazônia (INPA). Determination of functional groups was performed under different approaches. Resource removal strategy comprises a broad classification that commonly clusters dung beetle species according to their tribes and genera (Halffter and Edmonds 1982; Philips, Pretorius, and Scholtz 2004; Scholtz, Davis, and Kryger 2009). Nonetheless, diet is a highly specific classification, being strictly related to each species (Halffter and Halffter 2009; Scholtz, Davis, and Kryger 2009; Stavert et al. 2014). Through literature, we could extend the resource removal classification for the assemblage of the current study, but not the diet, due to the limited number of studies encompassing Amazonian dung beetles' diet (e.g., Edmonds and Zidek 2010; Ratcliffe 2013). To determine species' diet, we considered coprophagous or necrophagous species whenever more than 80% of their individuals were collected in excrement‐ or carrion‐baited traps, respectively (see Halffter and Arellano 2002). Species with less than 80% of their individuals collected in one of the resource types were classified as diet‐generalist. We determined species diet for those with *n* ≥ 5 captured individuals. Furthermore, dung beetles were classified into three groups according to their resource removal strategies: telecoprids (rollers), paracoprids (tunnelers), and endocoprids (dwellers) (Halffter and Edmonds 1982; Batilani‐Filho 2015).

To estimate species' body size, we measured the maximal pronotum width, which is often used as a proxy of body size (Emlem 1996; Kerman et al. 2018; Servín‐Pastor et al. 2020). Pronotum width was estimated using digital images obtained from a Samsung M32 digital camera under a microscope (Opton TIM‐2B). We analyzed photos in ImageJ software version 1.46r. To estimate species' body size from each species, we randomly used 30 beetles from the entire collection and then calculated the average. Whenever a species had less than 30 individuals captured, we used all collected specimens (based on Salomão et al. 2019; Correa, Ferreira, et al. 2021). By using a subset of the collected dung beetles, variations in species' size, which depend on sex and minor and major specimens' category (Emlem 1996; Scholtz, Davis, and Kryger 2009), could be accounted. All measurements were performed by the author Vanessa Pontes Mesquita. The collected materials were deposited in the entomological collections of INPA and the Universidade Federal do Mato Grosso (UFMT).

### 2.4 Landscape Metrics

Landscape cover variables were obtained from a categorical land‐cover map (MapBiomas Project—2017 Collection). The MapBiomas Amazon project is an initiative to contribute to the understanding of the transformations occurring in the Amazon territory through the annual mapping of land cover and land use in the Amazon territory, making the data freely available (Souza Jr et al. 2020). The MapBiomas project classifies and geo‐references landcovers using LANDSAT data for all Brazilian biomes at a 30‐m resolution with 95.9% accuracy level using an automatic classification routine (https://mapbiomas.org/analise‐de‐acuracia).

We quantified landscape structure variables that describe the composition and configuration of the landscapes around each sampling site: amount of forest (old growth and/or secondary vegetation in different stages of regeneration), urban, agriculture, pasture, and water cover, number of forest patches, and edge density. The metric number of forest patches describes the fragmentation of the landscape; however, this metric does not necessarily contain information about the configuration or composition of the landscape (Hesselbarth et al. 2019); it is estimated by identifying all nonconnected forest fragments counted within the buffer around each sampling site using the eight‐cell rule. Edge density, a configuration metric, is calculated as the length, in meters, of the forest edge contained within the buffer per ha (the boundary of the buffer that defines the landscape was not included in the total forest class edge length). For calculating landscape structure variables, we selected five different spatial scales (within a buffer radius of 200, 400, 600, 800, and 1000 m centered in each sampling site) to cover maximum home range size of beetle species (Cultid‐Medina et al. 2015; Barretto et al. 2021; Salomão, Arriaga‐Jiménez, and Kohlmann 2021). We used QGIS 3.20.1‐Odense software (QGIS 2021) for visualizing landcover maps and R package *landscapemetrics* for calculating landscape metrics using the categorical landscape maps in R software (Hesselbarth et al. 2019). Agriculture and pasture cover variables were grouped into the same cover class (hereafter agriculture–pasture cover class), and water cover was not further analyzed since this land cover category was present only around three sites sampled.

### 2.5 Data Analysis

We used generalized linear mixed models (GLMMs) with the template model builder (TMB) in the glmmTMB package (Brooks et al. 2017) to analyze the effects of landscape variables (forest cover, urban cover, agriculture–pasture cover, edge density, and number of forest patches) on dung beetles species richness (number of species recorded per sampling site), abundance (number of individuals recorded per sampling site), and body size. Body size per site comprised the average of species body size calculated for the species obtained in each site. The models incorporated the cities as random effects to account for potential spatial autocorrelations between sites within the same city. Response variables were analyzed considering the entire community and each functional group (according to resource removal strategy—tunnelers, rollers, and dwellers; and diet—diet generalists, coprophagous, and necrophagous) separately. As a first step, we used a scale‐selection procedure to select the best scale of landscape variables (circular buffers with 200, 400, 600, 800, and 1000 m of radius) for each response variable. This approach has been successfully used in other studies that encompass dung beetle assemblages in tropical forests (Salomão et al. 2019; Rivera, da Silva, and Favila 2021). The selection of regression models was based on Akaike's information criterion corrected for small sample size (AICc) in MuMln package (Bartón 2019). For each response variable, we performed a GLMM with each scale for each explanatory variable individually. We used the Poisson distribution for count variables species richness and abundance and the Gaussian distribution for continuous variable body mass. The best scale was those GLMM with ΔAICc ≤ 2.0 and Akaike weights (wi) closest to 1 (Burnham and Anderson 2004) (Tables S1–, S3). After selecting the best scale for each landscape variable, we performed a multivariate GLMM for each response variable. Before, we tested multicollinearity among the landscape variables using VIFcor function of usdm package (Naimi 2017). Forest cover and urban cover presented a high collinearity (*R* ^2^ > 0.75) for all GLMM models and thus we excluded urban cover from all models since we intend to evaluate how forest cover in urban areas influences the dung beetle community. The variance explained by the predictor variables of GLMMs (marginal variance—R^2^m—explained by the fixed variables, and conditional variance—R^2^c—explained by the entire model) was calculated using the r.squaredGLMM function in the MuMIn package. Analyses were performed using R software version 4.1.2 (R Development Core Team 2022).

To analyze the influence of forest cover, agriculture cover, edge density, and number of patches on dung beetle species composition, we performed the permutational multivariate analysis of variance (PERMANOVA) using Bray–Curtis dissimilarity index with adonis2 function in the vegan package (Oksanen et al. 2015). Species composition was assessed for the whole assemblage and functional groups according to the removal resource strategy and diet. Coprophagous, necrophagous, dwellers, and diet‐generalist beetles did not have enough samplings and thus were not analyzed in PERMANOVA. We estimated the PERMANOVA significance running 9999 permutations. We detect the scale effect as in PERMANOVA (ΔAICc ≤ 2.0; wi close to 1) using the fit_models function in the AICcPermanova package (Corcoran 2023, see Table S4).

We also performed similarity profile tests (SIMPROF) to explore the similarity in the dung beetle assemblage structure among the 38 sampling sites. SIMPROF was performed following Bray–Curtis similarity index. This approach forms cluster of samples (in our study, each study site) and assesses the statistical difference among the different clusters. SIMPROF compares the similarities between every pair of sampling sites, being such similarities ranked from smallest to largest. Similarities are analyzed among pairs of samples through their ranks of similarities (Clarke, Somerfield, and Gorley 2008). The statistically significant groups are structured according to a probability lower than 5% that they could be clustered randomly according to the data provided. Since forest cover at 600 m of radius was one of the scales that best explained shifts in dung beetle diversity (see Section 3 below), we included such variable in each sample unit (i.e., the 38 studied sites) of the SIMPROF results. SIMPROF was conducted with Primer software version 6.0 (Clarke and Gorley 2006).

## 3 Results

A total of 5576 dung beetles from 15 genera and 55 species were collected in the study sites (Table 1). *Canthon* Hoffmannsegg, 1817, and *Dichotomius* Hope, 1838, were the most diverse genera, with 8 and 7 species recorded, respectively, while *Pseudocanthon* Bates, 1887, *Phanaeus* MacLeay, 1819, and *Scybalocanthon* Martínez, 1948, were the least diverse genera, each one comprising two or one species. *Deltochilum submetallicum* species group, *Deltochilum aspericolle* species group, and *Canthon triangularis* (Drury, 1770) were the dominant species, together corresponding to 28% of the total abundance. In addition, these species were the most widespread, being recorded in 27 (*D. submetallicum* species group), 26 (*D. aspericolle* species group), and 22 ( *C. triangularis* ) of the 38 sites (Data S1). Species richness per city ranged from 36 to 28 species, being Manaus the most speciose city and Itacoatiara the least speciose one (Table 1). It is important to reinforce that eight sites were studied in Manaus and six sites were studied in each of the other cities. Regarding species abundance per city, Iranduba was the most abundant city (*n* = 1300), while Rio Preto da Eva was the least abundant (*n* = 530).

**TABLE 1:** Dung beetle species list, body size, resource removal strategy, and diet recorded in pitfall traps baited with carrion or excrement in 38 forest fragments located in six cities of Amazonas state, Brazil.

Species	Body size ± SD (mm)	Resource removal strategy	Diet	City name						Total
				Iranduba	Itacoatiara	Manacapuru	Manaus	Presidente Figueiredo	Rio Preto da Eva	
*Ateuchus* sp1	4.9 ± 0.03	Tun	Cop	19	98	0	0	0	0	117
*Ateuchus globulus*	6.5 ± 0.05	Tun	Cop	41	21	0	9	1	0	72
*Ateuchus murrayi*	5.0 ± 0.04	Tun	Cop	108	28	8	39	15	21	219
*Ateuchus simplex*	4.1 ± 0.11	Tun	Cop	39	0	0	6	0	0	45
*Ateuchus substriatum*	6.3 ± 0.12	Tun	Cop	0	0	12	7	0	0	19
*Canthidium deyrollei*	3.9 ± 0.11	Tun	Cop	9	0	4	73	0	0	86
*Canthidium lentum* species group	4.7 ± 0.11	Tun	Cop	184	2	49	7	1	2	245
*Canthidium* sp2	2.6 ± 0.02	Tun	Cop	0	7	0	0	0	0	7
*Canthidium* sp4	6.2 ± 0.12	Tun	Cop	30	0	18	95	52	19	214
*Canthon subcyanaeus*	5.6 ± 0.03	Rol	Cop	0	0	9	0	0	0	9
*Canthon xanthopus‐sericatus* species group	NA	Rol	Cop	0	4	0	0	0	0	4
*Canthon lituratus*	5.6 ± 0.04	Rol	Cop	0	0	11	0	0	0	11
*Canthon quadriguttatus*	5.0 ± 0.08	Rol	Cop	4	1	13	5	2	2	27
*Canthon sordidus*	8.1 ± 0.18	Rol	Nec	0	0	0	141	44	129	314
*Canthon* sp1	NA	Rol	NA	0	2	0	0	0	0	2
*Canthon* sp2	NA	Rol	Cop	4	0	0	0	0	0	4
*Canthon triangularis*	13.1 ± 0.13	Rol	Cop	3	181	3	110	7	16	320
*Coprophaneus jasius*	28 ± 0.32	Tun	Nec	14	6	5	39	78	54	196
*Coprophaneus lancifer*	33.7 ± 0.31	Tun	COP	1	3	2	10	5	16	37
*Deltochilum aspericolle* species group	7.8 ± 0.05	Rol	Nec	55	31	186	32	216	70	590
*Deltochilum carinatum* species group	NA	Rol	NA	0	0	0	1	0	0	1
*Deltochilum granulatum* species group	8.4 ± 0.04	Rol	Gen	18	19	2	27	173	63	302
*Deltochilum* sp1	NA	Rol	Cop	0	0	0	0	1	2	3
*Deltochilum submetallicum* species group	7.5 ± 0.06	Rol	Gen	134	33	235	26	221	38	687
*Dichotomius lucasi*	15.8 ± 0.12	Tun	Cop	26	8	12	105	3	11	165
*Dichotomius mamillatus*	24.1 ± 0.11	Tun	NA	0	0	0	7	0	0	7
*Dichotomius robustus*	21.6 ± 0.35	Tun	COP	18	0	13	2	0	1	34
*Dichotomius subanaeus*	NA	Tun	NA	0	0	0	0	3	0	3
*Dichotomius worontzowi*	21.9 ± 0.37	Tun	Cop	39	3	17	2	2	2	65
*Dichotomius boreus*	14.5 ± 1.60	Tun	Cop	19	83	10	68	7	5	192
*Dichotomius quadrilobatus*	15.4 ± 1.55	Tun	Cop	12	0	112	0	0	0	124
*Eurysternus atrosericus*	5.8 ± 0.05	Dwe	Cop	0	3	0	15	13	13	44
*Eurysternus caribaeus*	15.2 ± 0.52	Dwe	Cop	26	17	75	3	9	1	131
*Eurysternus cayannensis*	10.4 ± 0.04	Dwe	Cop	0	0	5	0	1	1	7
*Eurysternus hypocrita*	NA	Dwe	NA	0	0	0	2	1	1	4
*Eurysternus* sp1	7.6 ± 0.31	Dwe	Cop	0	0	7	3	1	3	14
*Eurysternus* sp2	9.9 ± 0.16	Dwe	Cop	0	0	19	3	4	0	26
*Ontherus appendiculatus*	11.6 ± 0.08	Tun	Cop	6	0	9	4	0	0	19
*Ontherus carinifrons*	17.1 ± 0.16	Tun	Cop	21	25	0	2	0	0	48
*Onthophagus rubrescens*	4.8 ± 0.09	Tun	Cop	9	101	14	16	17	5	162
*Onthophagus* sp2	5.1 ± 0.04	Tun	Cop	79	164	4	13	19	3	282
*Onthophagus* sp3	5.5 ± 0.08	Tun	Cop	0	16	0	0	1	0	17
*Oxysternon festivum*	17.6 ± 0.34	Tun	Cop	58	0	15	0	27	2	102
*Oxysternon lautum*	NA	Tun	NA	0	4	0	0	0	0	4
*Oxysternon silenus*	11.3 ± 0.11	Tun	Cop	0	0	0	1	16	7	24
*Phanaeus chalcomelas*	NA	Tun	NA	0	0	0	1	1	0	2
*Pseudocanthon xanthurus*	NA	Rol	NA	0	0	4	0	0		4
*Scybalocanthon pygidialis*	5.2 ± 0.03	Rol	Cop	0	3	0	0	0	6	9
*Scybalocanthon sexpilotus*	5.6 ± 0.05	Rol	Cop	0	15	0	1	0	0	16
*Sylvicanthon proseni*	8.2 ± 0.09	Rol	Cop	125	0	21	0	0	3	149
*Sylvicanthon seag*	6.6 ± 0.03	Rol	Cop	0	70	0	4	0	0	74
*Uroxys* sp1	2.8 ± 0.02	Tun	Cop	33	0	12	16	0	0	61
*Uroxys* sp2	2.8 ± 0.02	Tun	Cop	58	0	16	0	0	1	75
*Uroxys* sp3	3.5 ± 0.03	Tun	Cop	75	5	22	5	0	9	116
*Uroxys* sp4	2.2 ± 0.02	Tun	Cop	28	0	0	0	0	2	30
Abundance				1300	951	898	941	956	530	
Species richness				31	28	32	36	33	30	

*Note:* Abundance data were clustered according to city (each city comprised six studied fragments, except Manaus which had eight studied fragments. Tun—tunneler; Rol—roller; Dwe—dweller; Cop—coprophagous; Nec—necrophagous; Gen—diet generalist; NA—unavailable data). Species body size was obtained from a random set of 30 beetles of each species; whenever a species had *n* < 30, all individuals were used for this estimation.

*Coprophanaeus lancifer* (Linnaeus, 1767), *C. jasius* (Olivier, 1789), and *Dichotomius worontzowi* (Pereira, 1942) were the largest species, measuring 33.7, 28, and 21.9 mm, respectively, while *Uroxys* sp1, *Canthidium* sp2, and *Uroxys* sp4 were the smallest ones (respectively, measuring 2.8, 2.6, and 2.2 mm, see Table 1). A total of 4378 beetles from 54 species were collected in excrement‐baited traps and 1198 beetles from 37 species were collected in carrion‐baited pitfall traps. Regarding beetle diet, 36 species were classified as coprophagous, two as necrophagous (*D. aspericolle* species group, *C. jasius*), and two as diet‐generalists (*D. submetallicum* species group, *Deltochilum granulatum* species group). When considering resource removal strategies, most of the species were tunnelers (*s* = 31, *n* = 2825), followed by rollers (*s* = 18, *n* = 2526), being dwellers the least speciose (*s* = 6) and abundant (*n* = 225) group (Table 1).

**FIGURE 2:**
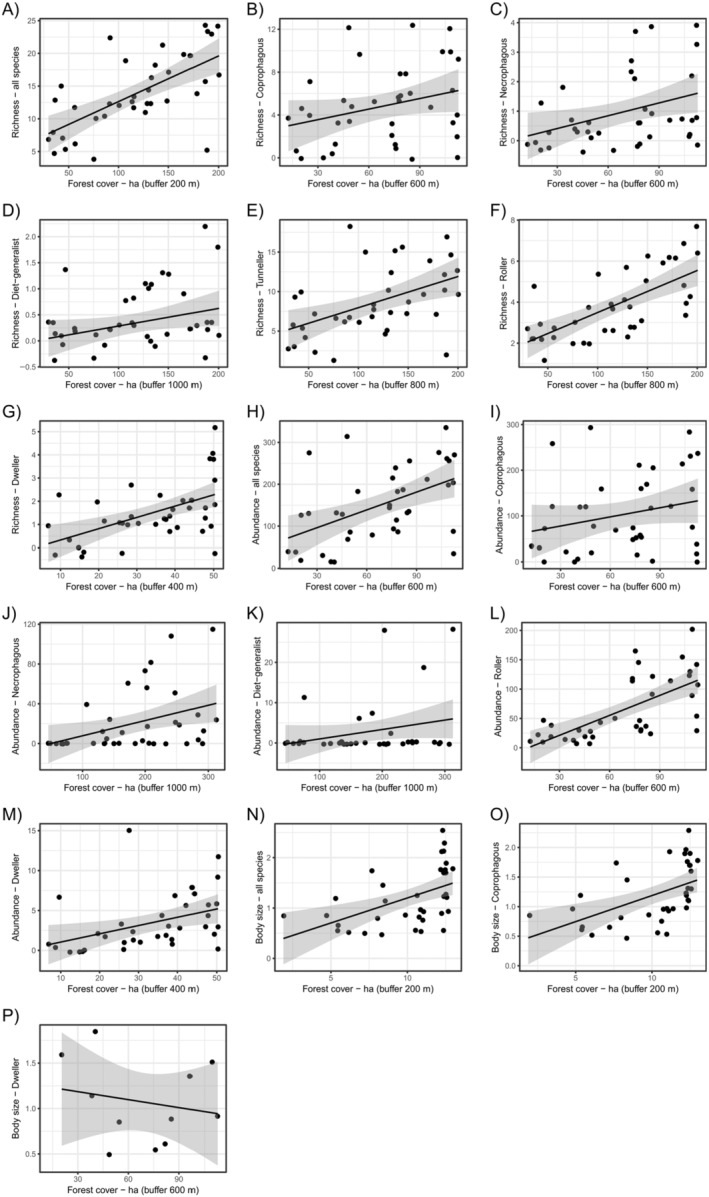
Linear regressions showing the statistically significant relationship between forest cover and dung beetle species richness, abundance, and body mass.

**FIGURE 3:**
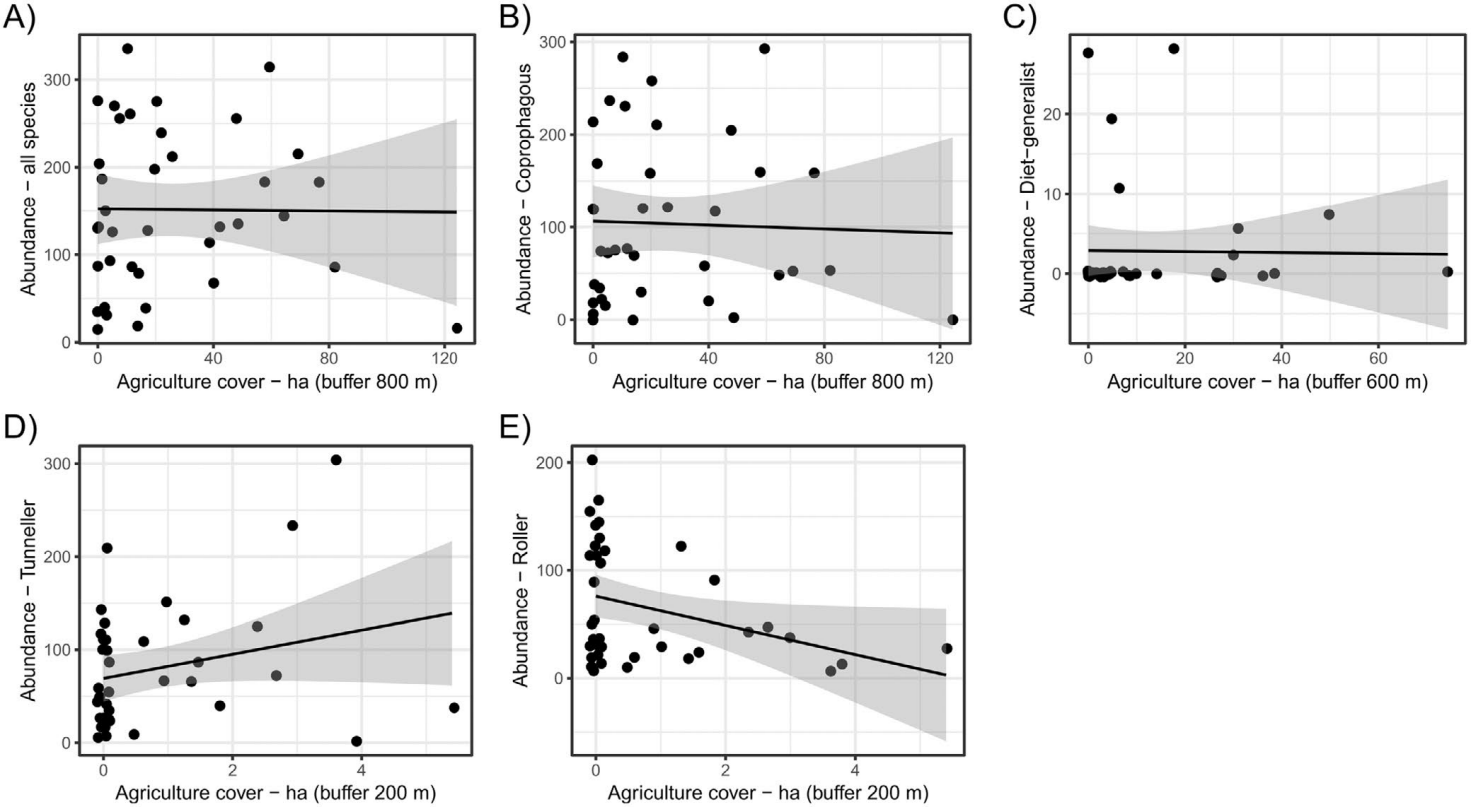
Linear regressions showing the statistically significant relationship between agriculture cover and dung beetle abundances.

**FIGURE 4:**
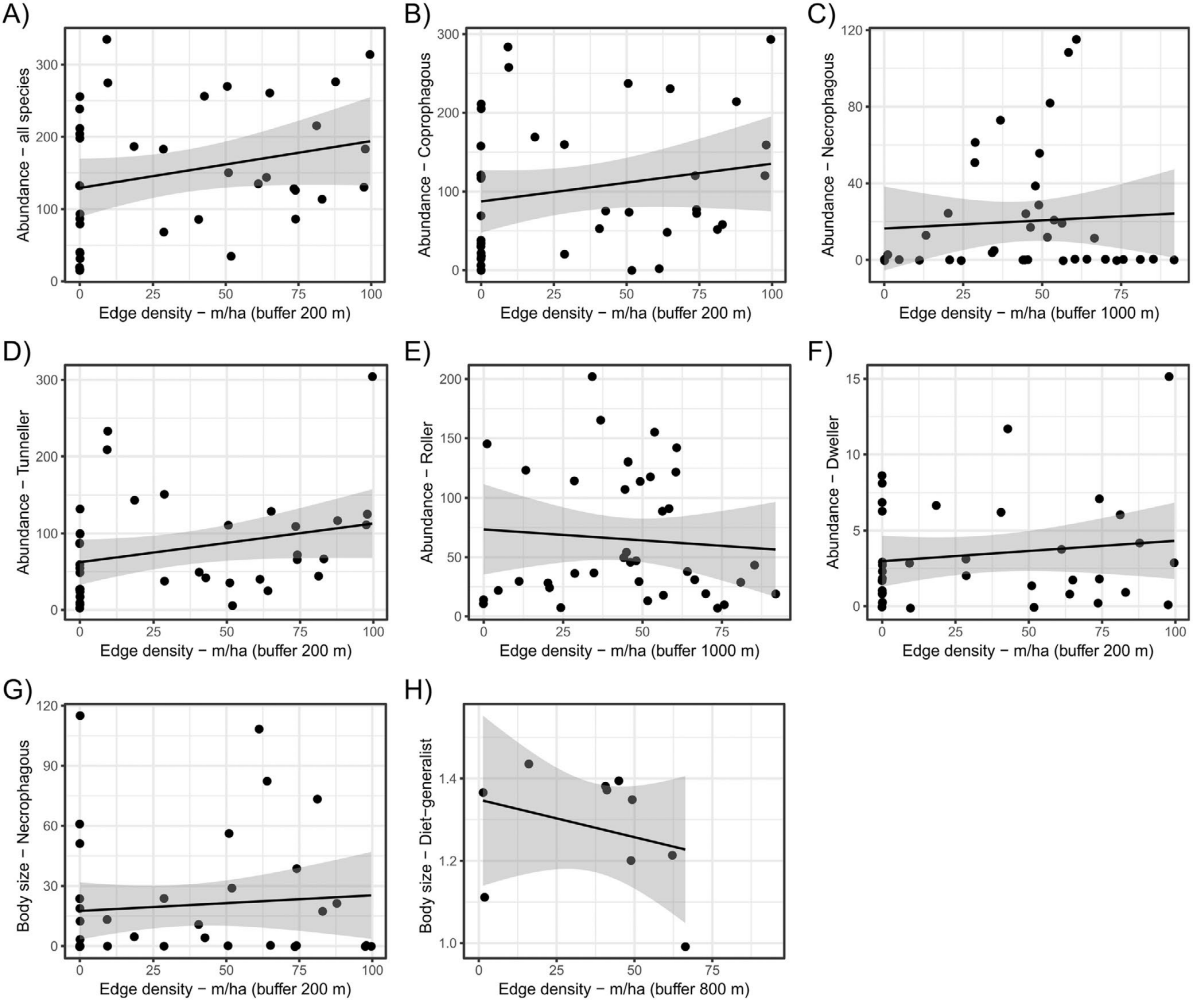
Linear regressions showing the significant relationship between edge density and dung beetle abundance and body mass.

**FIGURE 5:**
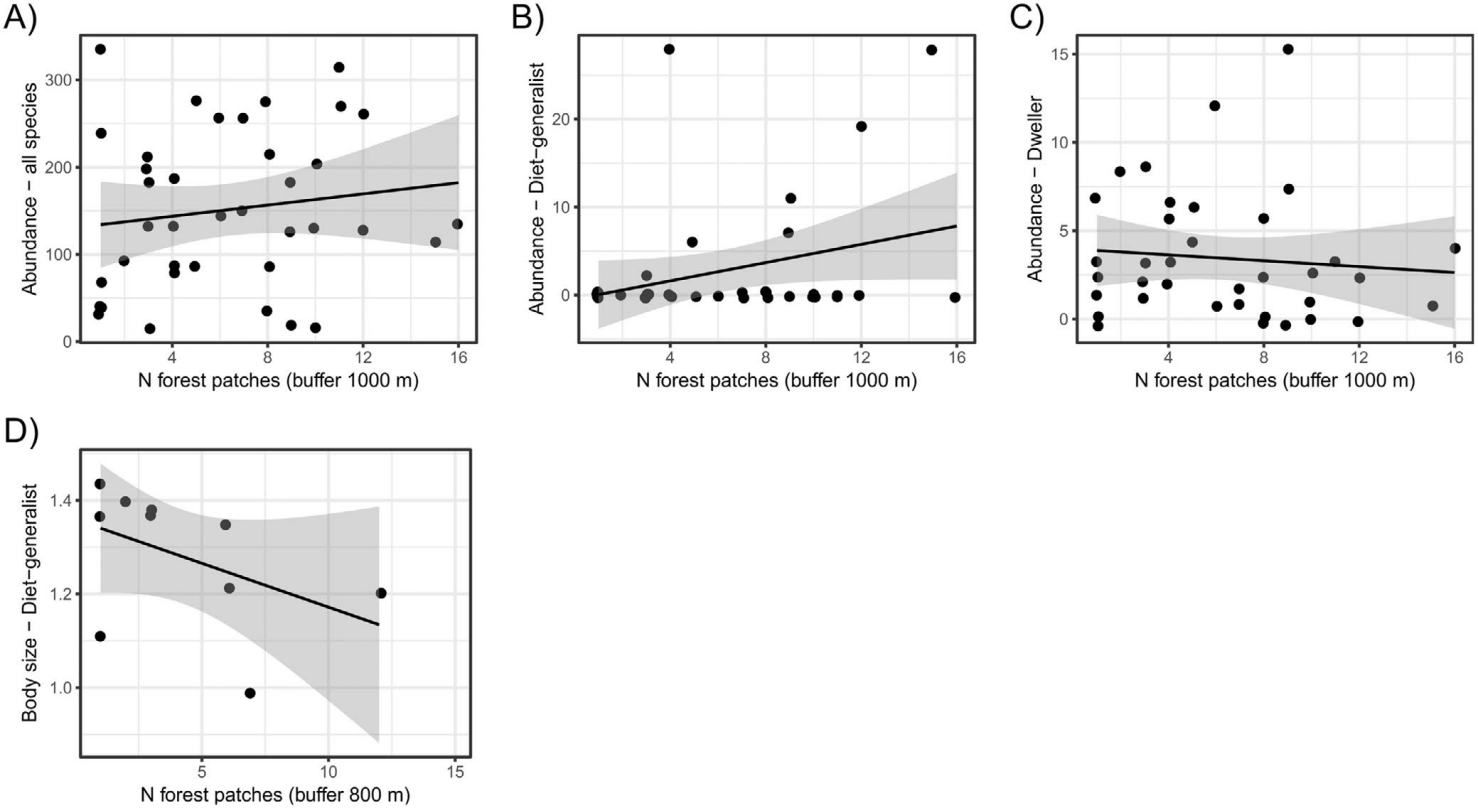
Linear regressions showing the significant relationship between the number of forest patches and dung beetle abundance and body mass.

Forest cover was the predictor variable that most affected beetle assemblage, presenting statistically significant relationships on species richness, abundance, and body size of the whole data, as well as for the different functional groups (Table 2). The increase in forest cover was positively related to dung beetle species richness (Figure 2A–G) and abundance (Figure 2H–M). Nonetheless, forest cover only affected the body size of the entire assemblage data, coprophagous species, presenting positive relationships (except for dwellers, which presented a positive relationship; Figure 2N–P). The amount of agricultural cover also affected dung beetle assemblage, although exclusively affecting their abundance (Table 2). Sites with higher amounts of agriculture cover comprised lower abundances of the entire dung beetle assemblage, coprophagous, and diet generalist species (Figure 3A–C). Tunneler and roller dung beetles showed opposite trend between them: While tunnelers increased their abundance in landscapes with larger agriculture cover (Figure 3D), rollers presented a negative relation with the increase in agriculture cover (Figure 3E). Edge density affected both dung beetle abundance and species' body size (Table 2). Landscapes with higher edge densities encompassed higher abundance of dung beetles (Figure 4). The only exceptions were the diet generalist and tunneler species—which were not affected by the edge density—and roller dung beetles, which had a negative relation with such landscape variables (Figure 4E). In addition, landscapes with higher edge densities maintained higher body size of necrophagous (Figure 4G), but lower body size of diet‐generalist species (Figure 4H). Lastly, the number of forest patches in the landscapes affected dung beetle abundance and body size (Table 2). Landscapes with higher number of forest patches maintained higher abundances of the entire assemblage of dung beetles as well as of diet‐generalist species (Figure 5A,B), but such pattern was opposite for dweller dung beetles, which decreased their abundances in landscapes with higher number of forest patches (Figure 5C). Landscapes with a higher number of forest patches also presented smaller body sizes for the diet‐generalist species (Figure 5D).

**TABLE 2:** Results of GLMM analysis of the effect of forest cover, agricultural cover, edge density, and number of patches on species richness, abundance, and body size of dung beetles for all species and for each species group (according to diet and resource removal strategy).

Response variable	Forest cover		Scale (m)	Agriculture cover		Scale (m)	Edge density		Scale (m)	N forest patches		Scale (m)	R^2^m	R^2^c
	*z*	*p*		*z*	*p*		*z*	*p*		*z*	*p*			
Richness														
All species	**5.76**	**< 0.01**	800	−0.70	0.48	800	1.07	0.28	600	−0.10	0.92	1000	0.49	0.58
Coprophagous	**2.41**	**0.01**	600	−1.03	0.30	800	1.84	0.066	200	0.56	0.58	200	0.15	0.64
Necrophagous	**2.34**	**0.02**	600	−0.36	0.72	200	0.41	0.68	600	−0.001	0.99	200	0.97	0.98
Tunneler	**4.11**	**< 0.01**	800	−0.94	0.34	800	1.86	0.06	200	−0.42	0.67	1000	0.33	0.56
Roller	**2.93**	**< 0.01**	800	0.23	0.82	200	0.78	0.44	1000	−0.82	0.42	600	0.27	0.27
Dweller	**2.96**	**< 0.01**	400	−0.03	0.97	400	−0.60	0.55	1000	−1.37	0.17	400	0.35	0.35
Diet‐generalist	**1.99**	**0.04**	1000	1.55	0.12	1000	−0.41	0.68	600	0.61	0.54	1000	0.27	0.27
Abundance														
All species	**21.60**	**< 0.01**	600	**−7.96**	**< 0.01**	800	**8.62**	**< 0.01**	200	**−2.81**	**< 0.01**	1000	0.60	0.97
Coprophagous	**10.10**	**< 0.01**	600	**−9.94**	**< 0.01**	800	**9.06**	**< 0.01**	200	−0.13	0.90	200	0.17	0.99
Necrophagous	**9.53**	**< 0.01**	1000	−1.18	0.24	200	**5.72**	**< 0.01**	1000	−0.003	0.99	200	0.98	0.99
Tunneler	0.31	0.76	200	**6.65**	**< 0.01**	200	**4.01**	**< 0.01**	200	−1.07	0.29	600	0.15	0.96
Roller	**21.81**	**< 0.01**	600	**−2.89**	**0.004**	200	**−3.32**	**< 0.01**	1000	1.26	0.21	600	0.78	0.97
Dweller	**4.21**	**< 0.01**	400	1.31	0.19	1000	**2.94**	**< 0.01**	200	**−2.70**	**< 0.01**	1000	0.57	0.57
Diet‐generalist	**5.34**	**< 0.01**	1000	**4.45**	**< 0.01**	600	0.96	0.34	600	**4.97**	**< 0.01**	1000	0.29	0.99
Body size														
All species	**3.02**	**< 0.01**	200	−0.22	0.83	200	−0.73	0.46	1000	0.02	0.98	800	0.28	0.28
Coprophagous	**2.86**	**< 0.01**	200	−0.20	0.84	200	0.49	0.63	1000	−1.35	0.18	800	0.32	0.32
Necrophagous	1.12	0.26	400	−0.97	0.33	200	**2.34**	**0.01**	200	0.71	0.48	1000	0.42	0.42
Tunneler	1.02	0.31	400	0.31	0.75	200	−1.19	0.23	600	1.37	0.17	400	0.10	0.11
Roller	1.07	0.29	200	−0.77	0.44	800	−0.10	0.92	1000	−0.14	0.89	800	0.11	0.11
Dweller	**−2.16**	**0.03**	600	−0.67	0.50	400	−1.12	0.26	200	1.40	0.16	400	0.31	0.78
Diet‐generalist	−0.49	0.63	600	0.33	0.73	200	**2.18**	**0.03**	800	**−3.41**	**0.001**	800	0.61	0.61

*Note:* Statistically significant effects are shown in bold. R^2^m = Marginal variance explained by the fixed variables; R^2^c = conditional variance explained by the entire model. The best effect scale was selected for each predictor variable.

**FIGURE 6:**
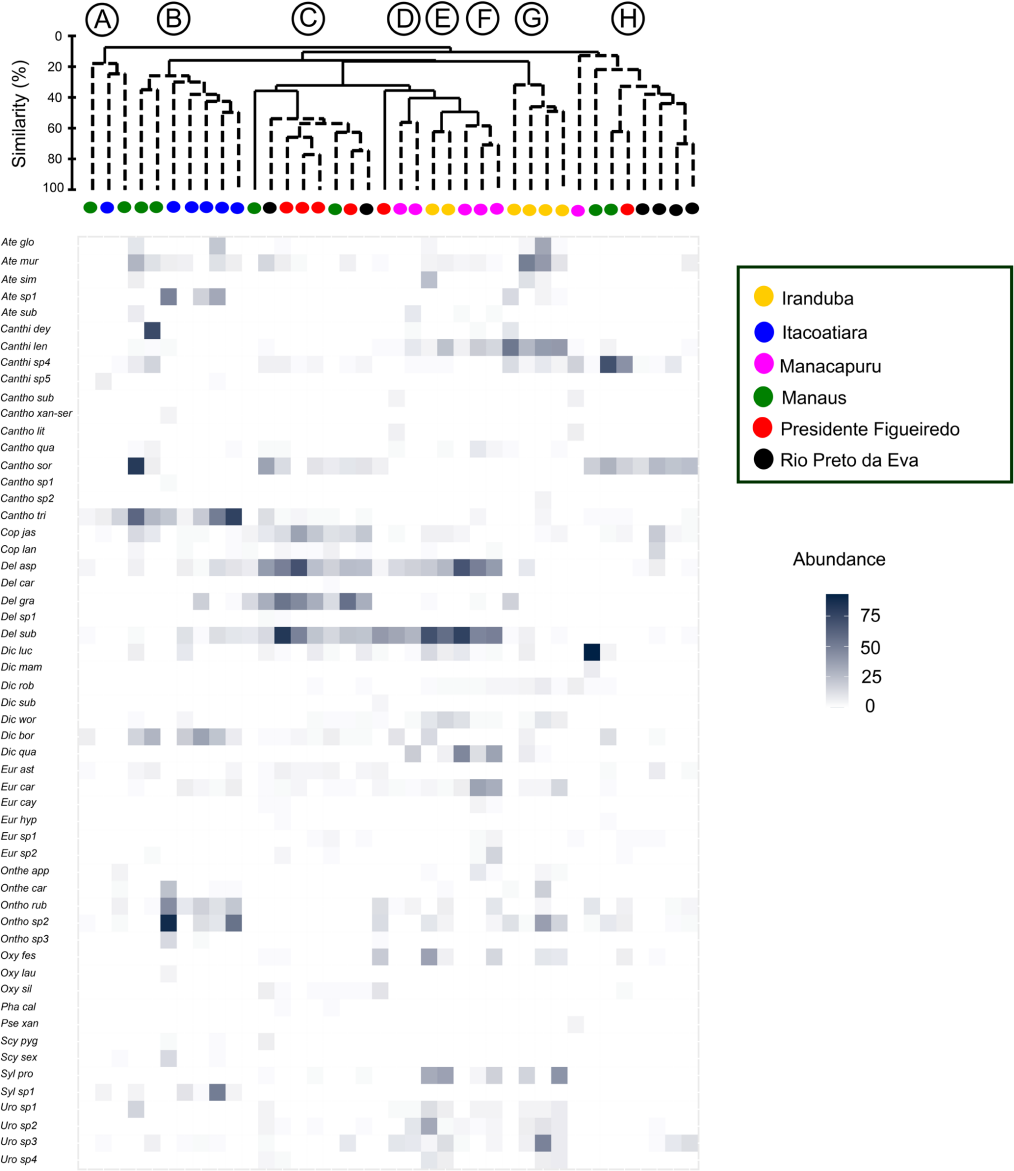
Heatmap showing species distribution of dung beetles recorded in 38 forest sites in six Amazonian cities with forest cover calculated at 600 m buffer scale. Dendrograms of group‐averaged clustering dung beetles and sampling sites using Bray–Curtis similarity index. Solid lines represent statistical differences between groups and dashed lines represent nonsignificant differences within groups (statistically significant groupings were presented by letters), based on SIMPROF analyses.

Forest cover was the only driver of differences in the dung beetle assemblage structure, affecting the entire assemblage data (Table 3) as well as the tunnelers and rollers species composition. Assemblage structure clustered dung beetles into eight significantly distinct groups (Figure 6). Group A comprised sites with low amount of forest cover from the two largest cities, Manaus (sites with 33 and 40 ha of forest cover at 600 m buffer scale) and Itacoatiara (20 ha of forest cover at 600 m buffer scale), clustering together with 17% similarity (Figure 6). The same was observed for sites with high amounts of forest cover from different cities, where group B comprised clusters from Manaus and Itacoatiara (25% similarity among such sites); group C comprised clusters from Rio Preto da Eva, Presidente Figueiredo, and Manaus (52%); group E from Iranduba (62%); and group F from Manacapuru (58%, Figure 6). The largest group (H, comprising 8 of the 38 studied fragments) grouped the sites with the lowest amounts of forest cover (with minimum of 12 ha and maximum 76 ha at 600 m buffer scale) from four of the six studied cities (Rio Preto da Eva, Manaus, Presidente Figueiredo, and Manacapuru, Figure 6), clustering together with 12% similarity.

**TABLE 3:** Results of PERMANOVA analysis of the effect of forest cover, agricultural cover, edge density, and number of patches on species composition of dung beetles for all species and for each species group (according to diet and resource removal strategy).

Response variable	Forest cover		Scale (m)	Agriculture cover		Scale (m)	Edge density		Scale (m)	N forest patches		Scale (m)
	*z*	*p*		*z*	*p*		*z*	*p*		*z*	*p*	
All species	**4.13**	**< 0.01**	800	1.26	0.22	200	1.19	0.28	800	1.22	0.23	800
Tunneler	**2.59**	**< 0.01**	400	1.04	0.42	200	1.02	0.42	1000	0.74	0.76	600
Roller	**5.78**	**< 0.01**	1000	0.82	0.59	200	1.13	0.34	1000	1.68	0.11	800

*Note:* Statistically significant effects are shown in bold. The best effect scale was selected for each predictor variable.

## 4 Discussion

In the Amazon cities of our study region, urban expansion had a relevant increase since the 80s, which was followed by forest loss and fragmentation of natural and seminatural ecosystems (Browder 2002; Chaves et al. 2021). The observed shifts in land use due to urbanization in this region, which is one of the most urbanized in Amazon (Barbieri 2007; Chaves et al. 2021), proved to be determinant for dung beetle assemblages. Among the results of this study, we emphasize that (1) forest cover was the main driver of shifts in dung beetle assemblage, while (2) agricultural cover, edge density, and number of patches affected mostly dung beetle abundances. These results show that shifts in ecological diversity caused by urbanization process are mostly driven by the amount of forest cover, while the other landscape variables related to configuration of the remaining forest influenced specific aspects of dung beetle assemblage.

### 4.1 Forest Cover as a Key Driver of Urbanization Effects on Amazonian Diversity

As observed in other taxonomic groups (Seress and Liker 2015; Hantak et al. 2021), forest cover was determinant for maintaining dung beetle biodiversity in urban landscapes. The loss of forest cover was the variable that most affected dung beetle assemblage structure, decreasing species richness, abundance, and body size and changing the species composition of the whole group and the different functional groups. In urban ecosystems, even small forest patches can act as a refuge for many forest‐dwelling species, while the urbanized matrix surrounding forest patches strongly limits their distribution and diversity (Korasaki et al. 2013; Kang et al. 2015; Zungu et al. 2020; Correa, Silva, et al. 2021). The decrease in forest cover has strongly affected the environmental conditions from the surrounding ecosystems towards the forest interior (Alvarado et al. 2018), as the increase in temperature and light incidence, and the reduction in humidity. Our results mirror the results of previous studies (Korasaki et al. 2013; Ramírez‐Restrepo and Halffter 2016; Salomão et al. 2019; Correa, Ferreira, et al. 2021), reinforcing the pattern that disturbances caused by the decrease of forest cover in urban landscapes impair the structure of dung beetle assemblages.

The results regarding body size and abundance presented in this study highlight a new and alarming scenario regarding urbanization in Amazonian urban landscapes. The observed decrease in abundance and body size with the decrease in forest cover suggests that there is limited availability and quality of food resources, which are key parameters that impair the establishment of higher abundances and large‐bodied individuals (Nichols et al. 2009; Zanette, Doyle, and Trémont 2000). Large‐bodied beetles tend to be more sensitive to environmental constraints (Hanski and Cambefort 1991; Scholtz, Davis, and Kryger 2009), as the decrease in its native habitat in urban ecosystems. Moreover, the loss of forest cover in cities is associated with the disappearance of native mammals (McKinney 2008; Villaseñor et al. 2014), which in turn have a direct relationship as resource providers for the dung beetles (Nichols et al. 2009). In other amazonian regions, diverse mammal species inhabit urban forest fragments (e.g., ocelot— *Leopardus pardalis* Linnaeus, 1758, opossum— *Didelphis marsupialis* Linnaeus, 1758; tamandua— *Myrmecophaga tridactyla* Linnaeus, 1758, Borges et al. 2014; Capaverde‐Jr et al. 2018), but it is important to consider that such information is lacking for the studied urban landscapes herein. Although there are non‐natural resources in the study sites analyzed herein (e.g., domestic animals' dung, decomposing organic material, and *personal observation*), we believe that such food types are of poor quality and little diverse because of the predominance of domestic animals in urban landscape compared to native animals' dung and carrion in conserved forests. The results presented herein indicate that the urbanization process in Amazon comes together with an oversimplification of ecological assemblages. The patterns observed herein directly impact the ecosystem services provided by the dung beetles (Nichols et al. 2008) and consequently the environmental stability in cities.

### 4.2 Effects of the Agriculture Cover, Amounts of Edge, and Number of Patches on Dung Beetle Diversity

In tropical forest regions, anthropogenic activities, including urban establishment and expansion, lead to habitat loss which in turn leads to habitat fragmentation with an increase in the amount of edges and the number of small and isolated forest patches (Püttker et al. 2020). In the urbanizing Amazonian landscapes studied here, landscapes with higher amounts of edges and number of forest patches supported higher abundance of dung beetles. This result was unexpected since the increase in both the amount of edges and number of patches is usually related to higher amounts of ecotones between tropical forests and anthropogenic matrices, increasing negative edge effect within forest fragments (Püttker et al. 2020). Although forest fragmentation challenges the maintenance of dung beetle assemblages (Nichols et al. 2007; Filgueiras et al. 2015), forest patches may also serve as stepping stones (Cultid‐Medina et al. 2015), which favor the populations in urban landscapes. The number of forest patches usually increases during the fragmentation process, however, until a certain point only, when the overall habitat amount is reduced, only a few small patches remain. Therefore, landscapes with a large number of patches (> 8 fragments in an area of 300 ha) may still not significantly lose connectivity because small patches act as stepping stones. These studies suggest that negative ecological effects are due to the dissimilarity between forest and the surrounding matrix and to the differences in habitat temperature. In the sites with the greatest density of edges, the species that most occurred among the tunneler group were *Canthidium* sp4, *C. jasius*, and *Dichotomius lucasi* (Harold, 1869). Despite the adverse conditions provided in sites with high edge density, the greater abundance of tunneler species may be related to the availability of environmental conditions established near the urban matrix. It is important to consider that edge density usually peaks at intermediate forest cover, which is because areas with small or large amount of forest cover have low amount of edges. Therefore, landscapes with large amount of edges maintain a certain amount of forest patches besides other types of urban environments and thus have a larger diversity of microenvironments. The abrupt edges established by urban fragments limit the distribution of most species. Among the abundant genera observed in sites with high density of edges, *Dichotomius* comprise species that are often abundant in non‐native conditions (Hernández and Vaz‐de‐Mello 2009). *Dichotomius lucasi*, *C. jasius*, and *Canthidium*, Erichson, 1847, species are widely distributed and highly abundant in native and non‐native habitats in Amazonian region (Scheffler 2005). The patterns found herein indicate that, although diversity of species is clearly depleted in more urbanized landscapes, certain landscape characteristics—such as the increase of edges and number of forest patches—can promote abundant assemblages. It is important to consider such patterns with care since the maintenance of abundant but poorly diversified assemblages impairs adequate ecosystem functioning (Nichols et al. 2008; Slade, Mann, and Lewis 2011).

Interestingly, agriculture cover only affected dung beetle abundances, presenting an overall negative effect, except for tunneler beetles, which were positively affected by the increase in agriculture cover. In tropical rainforests, agricultural lands can comprise limiting habits for native forest‐dweller dung beetles (Nichols et al. 2007; Filgueiras et al. 2015; Souza et al. 2020). Nonetheless, environmental conditions of agricultural lands seem to be milder when compared to urban habitats (Liu et al. 2010), which may explain the absence of effects of agriculture cover on dung beetle species richness and body size. Considering the decrease in spatial and trophic resources for forest‐dwelling dung beetles in landscapes with higher agricultural areas, it sounds plausible that consequently their populations would also be restrained. Regarding the positive effect of agriculture amount on tunneler abundance, we highlight that some of the recorded tunneler species have wide range distributions, inhabiting different regions of America (e.g., *Dichotomius boreus* and *Ontherus appendiculatus*) (Tissiani, Vaz‐de‐Mello, and Campelo‐Júnior 2017; Chamorro, Lopera‐Toro, and Rossini 2021). Tunnelers comprise the more speciose group among dung beetles and can exploit both conserved and disturbed habitats (e.g., Hanski and Cambefort 1991; Correa, Ferreira, et al. 2021). The subset of tunneler species that thrive under non‐native habitats, such as those with high amounts of agriculture land, could explain the positive relation between agriculture cover and tunneler abundance. The scenario observed herein suggests a different pattern of landscape effects on agricultural landscapes compared to more urbanized ones. Considering that matrix type can modulate the effects of forest loss and fragmentation on biodiversity, further studies could focus on disentangling how urban and nonurban matrices drive species spatial distribution in landscapes surrounded by agriculture.

We found that the landscapes with higher edge amounts and number of patches negatively affected diet‐generalists' body size and dweller abundances. It is important to analyze our results with care since the negative effects of the number of patches and edge amount observed on the dung beetles were punctual. In other words, we observed cues indicating limiting environmental conditions for dwellers (resulting in a decrease in their abundance). The number of forest patches usually increases during the fragmentation process; however, until a certain point only, when the overall habitat amount is reduced, only a few small patches remain. Therefore, landscapes with a large number of patches may still not significantly lose connectivity because small patches act as stepping stones. Our data show that the local imbalances caused by habitat loss and fragmentation of native habitats in tropical cities have different effects on the structure of dung beetle assemblages. It is important to carefully analyze the consequences of urban forest fragmentation on the above‐mentioned functional groups to determine the key aspects that allow the maintenance of healthy urban assemblages. Based on our results we recommend maintaining areas with intermediate amount of forest cover (at least 30%–40%) spread throughout the city, either as parks or linear green infrastructure. We highlight that small patches and edges are not necessarily negative for all dung beetle groups in an urban landscape context given an intermedia amount of forest cover. The complex set of effects related to forest fragmentation in this study reinforces the idea that different processes are co‐occurring and are determinants for the maintenance of biological diversity in cities.

### 4.3 Insights of Urbanization Effects on Biodiversity in the Tropics

Interestingly, the negative response of biodiversity to decrease in forest cover in cities of Amazon contrasts with previous research conducted in neighboring tropical rainforests (e.g., Salomão et al. 2019). In the Atlantic rainforest, located on almost the entire Brazilian coast, the decrease in forest cover in a city (João Pessoa, ca. 1,000,000 inhabitants) only affected specific subgroups of the dung beetle assemblage (Salomão et al. 2019). However, in the present study, the decrease in forest cover had a general negative effect on beetle assemblage. We suggest that João Pessoa dung beetle assemblages might be composed of generalist species, while the assemblages of the cities in this study may still present more sensitive species. It has been suggested that ecosystems that are well preserved maintain ecological communities that are more sensitive to land‐use transformation compared to those that have been affected by strong and chronic anthropogenic activities (Tocher 1998; Melo et al. 2013). In Brazil, the Atlantic rainforest has been undergoing an intense process of deforestation and urbanization since the beginning of the 16th century, and currently, there are only ca. 12% of the original vegetation (Santana, Delgado, and Schiavetti 2020; Ribeiro et al. 2011). In comparison, the Amazon has been experiencing a later process of modification of natural areas, which is related to the rapid and disorderly growth of cities (Barbieri, Monte‐Mór, and Bilsborrow 2009). Agricultural frontier development and deforestation in the Amazon follow the path of accelerated industrialization that began in the 1950s and amplified in Brazil's attempts to adapt to economic globalization (Vieira et al. 2008). In addition, the matrix that intersperses the Amazonian cities is mainly composed of forest areas, contrasting with the agricultural monoculture matrices that dominate other tropical ecosystems, such as the sugarcane, pastureland, and coffee landscapes of the Atlantic rainforest. Our results together with previous studies may indicate that forest loss effects on biodiversity in tropical cities are context dependent. We reinforce the findings presented by Sánchez‐de‐Jesús et al. (2016), which suggests that Amazon communities tend to be more sensitive to anthropogenic threats compared to communities of other tropical ecosystems. Future studies should focus on disentangling the role that ecosystem history (e.g., by using historical land use maps of the past decades) plays in the urbanization effects on biodiversity, by analyzing potential extinction debts in its current populations.

### 4.4 Study Limitations and Future Perspectives

Although our study had a robust sampling design that allowed us to properly attain its main goals, there were some issues that could broaden our perspectives in future studies. (1) The effects of urbanization on dung beetle body size were limited to assemblage‐scale perspectives; (2) spatial scale of landscape effects could be more deeply explored; and (3) dung beetle diversity potentially suffered from sampling gaps. While body traits can be analyzed by using mean values at assemblage scale (e.g., Salomão et al. 2019; Correa, Ferreira, et al. 2021)—as used herein—finer assessments can provide more detailed understanding of ecological processes on population patterns. Fine‐scale studies of individual traits (e.g., when analyzing each individual data) can present direct cause–consequence relationships between ecological processes, such as changes in environmental or nutritional availability and individual fitness and intraspecific competition (França et al. 2016; Servín‐Pastor et al. 2020). Regarding the landscape‐scale effects on biodiversity, novel studies are aiming to disentangle scales' role in understanding the landscape effects on biodiversity traits, which are strongly context dependent (Suárez‐Castro et al. 2018). In our study, species richness, abundance, and body size tended to represent a gradient from larger to smaller spatial scale regarding the scale with strongest effects on dung beetles. Moreover, from study that is currently being held by one of the coauthors of this study (RPS) in a savannah region of Brazil, higher orders of taxonomic diversity (hill numbers) present strongest response at larger spatial scales (RPS, unpublished data). These data suggest that assemblage traits present different levels of sensitivity, and we call special attention to body size data, which responded at small spatial landscape scales. This pattern could be related to the sensitivity of species body size toward fine environmental shifts (Nichols et al. 2007; Salomão et al. 2019). Lastly, due to the astonishing diversity and particular habits of dung beetles in Amazonian landscape (e.g., Larsen and Forsyth 2006; Salomão et al. 2024), this study could be suffering from potential gaps in the assessment of diversity in the urban fragments. Our study followed the most standardized methods to sample dung beetles (Iannuzzi et al. 2020; Mora‐Aguilar et al. 2023). However, particular techniques, such as the flying intercept trap, and particular methodological approaches, such as occupancy models, could shed some light on potential bias that diversity gaps could happen in the current study landscape. Albeit the current study is robust, the abovementioned issues can be used as ideas to be carefully analyzed in future studies encompassing landscape changes in tropical ecosystems.

## 5 Conclusion

In this study, we analyze how dung beetle communities respond to habitat loss and fragmentation of native forests in cities in the Amazon. Through this study, we provided important cues indicating that landscape composition and configuration strongly determine dung beetle diversity in cities located in the Amazon, the largest tropical rainforest in the world. Forest cover is featured as the most importance driving force of dung beetle assemblage. With the loss of forest cover, the decrease in beetles' body size and abundance directly affects ecological functions performed by them, such as nutrient cycling and soil aeration (Larsen, Lopera, and Forsyth 2008; Horgan 2008; Nichols et al. 2008). However, even in highly urbanized areas, landscapes with greater protected forest spaces provide a more balanced community. Even with the advance of deforestation and other anthropic activities, the Amazon cities still stand out among the tropical forests for having large portions of intact forest. In this sense, it is necessary to develop strategies that reconcile urban growth and the maintenance of this ecosystem's biodiversity. By assessing the effects of landscape transformation due to urbanization, we can have cues regarding the best distribution of land uses that will allow the proper maintenance of the urban ecosystems. Regarding the conservation dilemma of single large or several small fragments (Arroyo‐Rodríguez et al. 2020), our results indicate that large forest patches would be the best alternative for biodiversity conservation in urban Amazon scenario observed herein. Considering the importance of public policies aimed at urban growth in an orderly and planned way, future studies should consider the importance of parks, squares, and corridors as elements that can favor the maintenance of biodiversity and, consequently, a better quality of life for humans.

## Author Contributions

**Vanessa Pontes Mesquita:** data curation (lead), formal analysis (equal), investigation (lead), methodology (supporting), writing – original draft (equal). **Glenda Vanessa dos Santos Bernardino:** data curation (equal), methodology (equal), validation (equal), writing – original draft (supporting). **Paulo Estefano Dineli Bobrowiec:** data curation (supporting), formal analysis (lead), supervision (equal), writing – original draft (equal). **Renato Portela Salomão:** conceptualization (lead), data curation (equal), formal analysis (equal), investigation (lead), methodology (lead), project administration (lead), supervision (equal), writing – original draft (equal). **Cintia Cornelius:** conceptualization (lead), formal analysis (equal), funding acquisition (lead), investigation (supporting), project administration (lead), resources (lead), supervision (equal), writing – original draft (equal).

## Conflicts of Interest

The authors declare no conflicts of interest.

### Open Research Badges

This article has earned an Open Data badge for making publicly available the digitally‐shareable data necessary to reproduce the reported results. The data is available at [https://osf.io/tvyxz/wiki/2.%20Awarding%20Badges/].

## Supporting information


Data S1.

Table S1.

Table S2.

Table S3.

Table S4.

## Data Availability

The data that support the findings of this study are available in the Supporting Information of this article.

## References

[ece370704-bib-0001] Alvarado, F. , E. R. Andrade , B. A. Santos , G. Prescott , G. Souza , and F. Escobar . 2018. “Forest Cover is More Important Than Farmland Heterogeneity and Livestock Intensification for the Retention of Dung Beetle Phylogenetic Diversity.” Ecological Indicators 93: 524–532. 10.1016/j.ecolind.2018.05.041.

[ece370704-bib-0002] Aronson, M. F. , F. A. La Sorte , C. H. Nilon , et al. 2014. “A Global Analysis of the Impacts of Urbanization on Bird and Plant Diversity Reveals Key Anthropogenic Drivers.” Proceedings of the Royal Society B: Biological Sciences 281, no. 1780: 20133330. 10.1098/rspb.2013.3330. PMC4027400 24523278

[ece370704-bib-0003] Arroyo‐Rodríguez, V. , L. Fahrig , M. Tabarelli , et al. 2020. “Designing Optimal Human‐Modified Landscapes for Forest Biodiversity Conservation.” Ecology Letters 239: 1404–1420. 10.1111/ele.13535. 32537896

[ece370704-bib-0004] Barbieri, A. F. 2007. “Mobilidade Populacional, Meio Ambiente e Uso da Terra Em Áreas de Fronteira: Uma Abordagem Multiescalar.” Revista Brasileira de Estudos de População 24: 225–246. 10.1590/S0102-30982007000200004.

[ece370704-bib-0005] Barbieri, A. F. , R. L. Monte‐Mór , and R. E. Bilsborrow . 2009. “Urban Population‐Environment Dynamics in the Developing World: Case Studies and Lessons Learned.” In Towns in the Jungle: Exploring Linkages Between Rural–Urban Mobility, Urbanization and Development in the Amazon, edited by A. F. Barbieri , R. L. M. Monte‐Mor , and R. E. Bilsborrow , 247–279. Paris, France: Committee for International Cooperation in National Research in Demography.

[ece370704-bib-0006] Barretto, J. , M. L. Baena , I. H. Domínguez , and F. Escobar . 2021. “Spatiotemporal Variation in the Adult Sex Ratio, Male Aggregation, and Movement of Two Tropical Cloud Forest Dung Beetles.” Current Zoology 68, no. 6: 635–644. 10.1093/cz/zoab101. 36743229 PMC9892795

[ece370704-bib-0113] Bartón, K. 2019. MuMIn: Multi‐Model Inference. R package.

[ece370704-bib-0007] Batilani‐Filho, M. 2015. “Funções Ecossistêmicas Realizadas por Besouros Scarabaeinae na Decomposição da Matéria Orgânica: Aspectos Quantitativos em Áreas de Mata Atlântica.” Dissertation, Universidade Federal de Santa Catarina.

[ece370704-bib-0110] Bernardino, G. V. S. , V. P. Mesquita , P. E. D. Bobrowiec , L. Iannuzzi , R. P. Salomão , and C. Cornelius . 2024. “Habitat Loss Reduces Abundance and Body Size of Forest‐Dwelling Dung Beetles in an Amazonian Urban Landscape.” Urban Ecosystems 27, no. 4: 1175–1190. 10.1007/s11252-024-01520-6.

[ece370704-bib-0008] Borges, L. H. M. , A. M. Calouro , A. L. M. Botelho , and M. Silveira . 2014. “Diversity and Habitat Preference of Medium and Large‐Sized Mammals in Na Urban Forest Fragment of Southwestern Amazon.” Iheringia Serie Zoologica 104, no. 2: 168–174. 10.1590/1678-476620141042168174.

[ece370704-bib-0009] Brooks, M. E. , K. Kristensen , K. J. van Benthem , A. Magnusson , C. W. Berg , and A. Nielsen . 2017. “Generalized Linear Mixed Models Using Template Model Builder.” R package.

[ece370704-bib-0010] Browder, J. O. 2002. “The Urban–Rural Interface: Urbanization and Tropical Forest Cover Change.” Urban Ecosystems 6, no. 1: 21–41. 10.1023/A:1025962512653.

[ece370704-bib-0011] Burnham, K. P. , and D. R. Anderson . 2004. “Multimodel Inference: Understanding AIC and BIC in Model Selection.” Sociological Methods & Research 33, no. 2: 261–304. 10.1177/00491241042686.

[ece370704-bib-0012] Capaverde‐Jr, U. D. , M. S. Lopes , N. C. V. Almeida , F. Z. P. Almeida , and D. B. Pathek . 2018. “Wild Animals Collected by the Independent Company of Environmental Police Monte Roraima in Urban Area of Boa Vista, Brazilian Amazon.” Biota Amazônica 8: 43–48. 10.18561/2179-5746/biotaamazonia.v8n1p43-48.

[ece370704-bib-0013] Carvalho, R. L. , A. N. Andersen , D. V. Anjos , R. Pacheco , L. Chagas , and H. L. Vasconcelos . 2020. “Understanding What Bioindicators are Actually Indicating: Linking Disturbance Responses to Ecological Traits of Dung Beetles and Ants.” Ecological Indicators 108: 105764. 10.1016/j.ecolind.2019.105764.

[ece370704-bib-0014] Chamorro, W. , A. Lopera‐Toro , and M. Rossini . 2021. “A New Species and Distribution Records of *Dichotomius* Hope, 1838 (Coleoptera: Scarabaeidae: Scarabaeinae) in Colombia.” Zootaxa 4942, no. 2: 193–206. 10.11646/zootaxa.4942.2.3. 33757065

[ece370704-bib-0015] Chaves, W. A. , D. Valle , A. S. Tavares , T. Q. Morcatty , and D. S. Wilcove . 2021. “Impacts of Rural to Urban Migration, Urbanization, and Generational Change on Consumption of Wild Animals in the Amazon.” Conservation Biology 354: 1186–1197. 10.1111/cobi.13663. 33124717

[ece370704-bib-0016] Clarke, K. R. , and R. N. Gorley . 2006. Primer v6 User Manual/Tutorial. Plymouth, UK: Primer‐E Ltd.

[ece370704-bib-0017] Clarke, K. R. , P. J. Somerfield , and R. N. Gorley . 2008. “Testing of Null Hypotheses in Exploratory Community Analyses: Similarity Profiles and Biota‐Environment Linkage.” Journal of Experimental Marine Biology and Ecology 366: 56–69. 10.1016/j.jembe.2008.07.009.

[ece370704-bib-0114] Corcoran, D. 2023. AICcPermanova: Model Selection of PERMANOVA Models Using AICc. R package.

[ece370704-bib-0018] Correa, C. , K. R. Ferreira , A. Puker , L. D. Audino , and V. Korasaki . 2021. “Greenspace Sites Conserve Taxonomic and Functional Diversity of Dung Beetles in an Urbanized Landscape in the Brazilian Cerrado.” Urban Ecosystems 245: 1023–1034. 10.1007/s11252-021-01093-8.

[ece370704-bib-0019] Correa, C. M. , P. G. Silva , M. A. Lara , and A. Puker . 2021. “Spatiotemporal Patterns of β‐Diversity of Flower Chafer Beetles in Urban Park and Natural Reserve Sites in Brazilian Cerrado.” International Journal of Tropical Insect Science 411: 681–691. 10.1007/s42690-020-00257-x.

[ece370704-bib-0020] Costa, J. R. , L. M. L. Colares , and G. R. Monteiro . 2022. “Caracterização da Flora e da Fauna em Estudo Ambiental Simplificado na Amazônia Central.” Biodiversidade 21, no. 3: 72–88.

[ece370704-bib-0021] Cultid‐Medina, C. A. , B. G. Martínez‐Quintero , F. Escobar , and P. C. de Ulloa . 2015. “Movement and Population Size of Two Dung Beetle Species in an Andean Agricultural Landscape Dominated by Sun‐Grown Coffee.” Journal of Insect Conservation 19, no. 4: 617–626. 10.1007/s10841-015-9784-3.

[ece370704-bib-0022] Cupello, M. , and F. Z. Vaz‐de‐Mello . 2019. “A Monographic Revision of the Neotropical Dung Beetle Genus *Sylvicanthon* Halffter & Martínez, 1977 (Coleoptera: Scarabaeidae: Scarabaeinae: Deltochilini), Including a Reappraisal of the Taxonomic History of ‘Canthon Sensu Lato’.” European Journal of Taxonomy 467: 1–205. 10.5852/ejt.2018.467.

[ece370704-bib-0023] Deng, C. , D. Zhu , X. Nie , et al. 2021. “Precipitation and Urban Expansion Caused Jointly the Spatiotemporal Dislocation Between Supply and Demand of Water Provision Service.” Journal of Environmental Management 29, no. 9: 113–660. 10.1016/j.jenvman.2021.113660. 34481371

[ece370704-bib-0024] Edmonds, W. D. , and J. Zidek . 2010. “A Taxonomic Review of the Neotropical Genus Coprophanaeus Olsoufieff, 1924 (Coleoptera: Scarabaeidae, Scarabaeinae).” Insecta Mundi 129: 1–112.

[ece370704-bib-0025] Emlem, D. J. 1996. “Artificial Selection on Horn Length‐Body Size Allometry in the Horned Beetle *Onthophagus acuminatus* (Coleoptera: Scarabaeidae).” Evolution 50: 1219–1230. 10.2307/2410662. 28565264

[ece370704-bib-0026] Filgueiras, B. K. C. , M. Tabarelli , I. R. Leal , F. Z. Vaz‐de‐Mello , and L. Iannuzzi . 2015. “Dung Beetle Persistence in Human‐Modified Landscapes: Combining Indicator Species With Anthropogenic Land Use and Fragmentation‐Related Effects.” Ecological Indicators 55: 65–73. 10.1016/j.ecolind.2015.02.032.

[ece370704-bib-0027] França, F. , J. Barlow , B. Araújo , and J. Louzada . 2016. “Does Selective Logging Stress Tropical Forest Invertebrates? Using Fat Stores to Examine Sublethal Responses in Dung Beetles.” Ecology and Evolution 6, no. 23: 8526–8533. 10.1002/ece3.2488. 28031804 PMC5167030

[ece370704-bib-0028] Gardner, T. A. , M. I. Hernández , J. Barlow , and C. A. Peres . 2008. “Understanding the Biodiversity Consequences of Habitat Change: The Value of Secondary and Plantation Forests for Neotropical Dung Beetles.” Journal of Applied Ecology 45, no. 3: 883–893. 10.1111/j.1365-2664.2008.01454.x.

[ece370704-bib-0029] Génier, F. 2009. Le Genre Eurysternus Dalman, 1824 (Scarabaeidae: Scarabaeinae: Oniticellini), Révision Taxonomique et Clés de Détermination Illustrées, 430. Sofía‐Moscow: Pensoft.

[ece370704-bib-0030] Halffter, G. , and L. Arellano . 2002. “Response of Dung Beetle Diversity to Human–Induced Changes in a Tropical Landscape 1.” Biotropica 34, no. 1: 144–154. 10.1111/j.1744-7429.2002.tb00250.x.

[ece370704-bib-0031] Halffter, G. , and W. D. Edmonds , eds. 1982. “The Nesting Behavior of Dung Beetles (Scarabaeinae).” In The Nesting Behavior of Dung Beetles (Scarabaeinae). An Ecological and Evolutive Approach, 176. México, D.F: Instituto de Ecología.

[ece370704-bib-0111] Halffter, G. , and V. Halffter . 2009. “Why and Where Coprophagous Beetles (Coleoptera: Scarabaeinae) Eat Seeds, Fruits or Vegetable Detritus.” Boletín Sociedad Entomológica Aragonesa 45: 1–22.

[ece370704-bib-0032] Hanski, I. , and Y. Cambefort . 1991. Dung Beetle Ecology. Princeton, NJ: Princeton University Press.

[ece370704-bib-0033] Hantak, M. M. , B. S. McLean , D. Li , and R. P. Guralnick . 2021. “Mammalian Body Size is Determined by Interactions Between Climate, Urbanization, and Ecological Traits.” Communications Biology 4, no. 1: 972. 10.1038/s42003-021-02505-3. 34400755 PMC8367959

[ece370704-bib-0034] Harper, K. A. , S. E. Macdonald , P. J. Burton , et al. 2005. “Edge Influence on Forest Structure and Composition in Fragmented Landscapes.” Conservation Biology 193: 768–782. 10.1111/j.1523-1739.2005.00045.x.

[ece370704-bib-0035] Hernández, M. I. M. , and F. Z. Vaz‐de‐Mello . 2009. “Seasonal and Spatial Species Richness Variation of Dung Beetle (Coleoptera, Scarabaeidae s. Str.) in the Atlantic Forest of Southeastern Brazil.” Revista Brasileira de Entomologia 53: 607–613. 10.1590/S0085-56262009000400010.

[ece370704-bib-0036] Hesselbarth, M. H. K. , M. Sciaini , K. A. With , K. Wiegand , and J. Nowosad . 2019. “Landscapemetrics: An Open‐Source R Tool to Calculate Landscape Metrics.” Ecography 42: 1648–1657. 10.1111/ecog.04617.

[ece370704-bib-0037] Horgan, F. G. 2008. “Dung Beetle Assemblages in Forests and Pastures of El Salvador: A Functional Comparison.” Biodiversity and Conservation 17: 2961–2978. 10.1007/s10531-008-9408-2.

[ece370704-bib-0038] Iannuzzi, L. , C. N. Liberal , T. B. Souza , et al. 2020. “Sampling Methods for Beetles (Coleoptera).” In Measuring Arhtropod Diversity, edited by J. C. Santos and G. W. Fernandes , 125–185. Cham, Switzerland: Springer.

[ece370704-bib-0039] Iannuzzi, L. , R. P. Salomão , F. C. Costa , and C. N. Liberal . 2016. “Environmental Patterns and Daily Activity of Dung Beetles (Coleoptera: Scarabaeidae) in the Atlantic Rainforest of Brazil.” Entomotropica 31: 196–207.

[ece370704-bib-0040] Instituto Brasileiro de Geografia e Estatística – IBGE . 2022. “Estimativas da População Residente No Brasil e Unidades da Federação Com Data de Referência Em 1° de Julho de 2021.” https://ftp.ibge.gov.br/Estimativas_de_Populacao/Estimativas_2021/estimativa_dou_2021.pdf.

[ece370704-bib-0041] Instituto Nacional de Meteorologia do Brasil – INMET . 2011. “Normas Climatológicas (1961/1990).”

[ece370704-bib-0042] Kang, W. , E. S. Minor , C. R. Park , and D. Lee . 2015. “Effects of Habitat Structure, Human Disturbance, and Habitat Connectivity on Urban Forest Bird Communities.” Urban Ecosystems 183: 857–870. 10.1007/s11252-014-0433-5.

[ece370704-bib-0043] Kerman, K. , A. Roggero , A. Rolando , and C. Palestrini . 2018. “Evidence for Male Horn Dimorphism and Related Pronotal Shape Variation in *Copris lunaris* (Linnaeus, 1758) (Coleoptera: Scarabaeidae, Coprini).” Insects 9: 108. 10.3390/insects9030108. 30135396 PMC6164466

[ece370704-bib-0044] Korasaki, V. , J. Lopes , G. Gardner‐Brown , and J. Louzada . 2013. “Using Dung Beetles to Evaluate the Effects of Urbanization on Atlantic Forest Biodiversity.” Insect Science 203: 393–406. 10.1111/j.1744-7917.2012.01509.x. 23955891

[ece370704-bib-0045] Kowarik, I. 2011. “Novel Urban Ecosystems, Biodiversity, and Conservation.” Environmental Pollution 1598‐9: 1974–1983. 10.1016/j.envpol.2011.02.022. 21435761

[ece370704-bib-0046] Larsen, T. H. , and A. Forsyth . 2006. “Extreme Trophic and Habitat Specialization by Peruvian Dung Beetles (Coleoptera: Scarabaeidae: Scarabaeinae).” Coleopterists Bulletin 60, no. 4: 315–324. 10.1649/0010-065X(2006)60[315:ETAHSB]2.0.CO;2.

[ece370704-bib-0047] Larsen, T. H. , A. Lopera , and A. Forsyth . 2008. “Understanding Trait‐Dependent Community Disassembly: Dung Beetles, Density Functions, and Forest Fragmentation.” Conservation Biology 225: 1288–1298. 10.1111/j.1523-1739.2008.00969.x. 18616744

[ece370704-bib-0048] Laurance, W. , H. Vasconcelos , and T. Lovejoy . 2000. “Forest Loss and Fragmentation in the Amazon: Implications for Wildlife Conservation.” Oryx 34, no. 1: 39–45. 10.1046/j.1365-3008.2000.00094.x.

[ece370704-bib-0049] Laurance, W. F. , M. Goosem , and S. G. Laurance . 2009. “Impacts of Roads and Linear Clearings on Tropical Forests.” Trends in Ecology & Evolution 24, no. 12: 659–669. 10.1016/j.tree.2009.06.009. 19748151

[ece370704-bib-0050] Levis, C. , F. R. Costa , F. Bongers , et al. 2017. “Persistent Effects of Pre‐Columbian Plant Domestication on Amazonian Forest Composition.” Science 355, no. 6328: 925–931. 10.1126/science.aal0157. 28254935

[ece370704-bib-0051] Liu, W. , J. Zhan , F. Zhao , H. Yan , F. Zhang , and X. Wei . 2019. “Impacts of Urbanization‐Induced Land‐Use Changes on Ecosystem Services: A Case Study of the Pearl River Delta Metropolitan Region, China.” Ecological Indicators 98: 228–238. 10.1016/j.ecolind.2018.10.054.

[ece370704-bib-0052] Liu, Y. , J. C. Axmacher , C. Wang , L. Li , and Z. Yu . 2010. “Ground Beetles (Coleoptera: Carabidae) in the Intensively Cultivated Agricultural Landscape of Northern China–Implications for Biodiversity Conservation.” Insect Conservation and Diversity 3, no. 1: 34–43. 10.1111/j.1752-4598.2009.00069.x.

[ece370704-bib-0053] Macedo, M. V. A. , and E. E. Filippi . 2021. A Amazônia e a Sua Progressiva Destruição Florestal Pela Ação Antrópica. Fórum Internacional Ecoinovar (10.: 2021:Online). Anais. Santa Maria, CA: UFSM.

[ece370704-bib-0054] Magura, T. , M. Ferrante , and G. L. Lövei . 2020. “Only Habitat Specialists Become Smaller With Advancing Urbanization.” Global Ecololgy and Biogeography 29: 1978–1987. 10.1111/geb.13168.

[ece370704-bib-0055] McKinney, M. L. 2002. “Urbanization, Biodiversity, and Conservation: The Impacts of Urbanization on Native Species are Poorly Studied, but Educating a Highly Urbanized Human Population About These Impacts Can Greatly Improve Species Conservation in All Ecosystems.” Bioscience 210: 883–890. 10.1641/0006-3568(2002)052[0883:UBAC]2.0.CO;2.

[ece370704-bib-0056] McKinney, M. L. 2008. “Effects of Urbanization on Species Richness: A Review of Plants and Animals.” Urban Ecosystems 112: 161–176. 10.1007/s11252-007-0045-4.

[ece370704-bib-0057] Melo, F. P. , V. Arroyo‐Rodríguez , L. Fahrig , M. Martínez‐Ramos , and M. Tabarelli . 2013. “On the Hope for Biodiversity‐Friendly Tropical Landscapes.” Trends in Ecology & Evolution 28, no. 8: 462–468. 10.1016/j.tree.2013.01.001. 23375444

[ece370704-bib-0058] Metzger, J. P. 2001. “O que é ecologia de paisagens?” Biota Neotropica 1, no. 1/2: 1–9. 10.1590/S1676-06032001000100006.

[ece370704-bib-0059] Mora‐Aguilar, E. F. , A. Arriaga‐Jiménez , C. M. A. Correa , et al. 2023. “Toward a Standardized Methodology for Sampling Dung Beetles (Coleoptera: Scarabaeinae) in the Neotropics: A Critical Review.” Frontiers in Ecology and Evolution 11: 1096208. 10.3389/fevo.2023.1096208.

[ece370704-bib-0060] Müller, A. , P. K. Bøcher , C. Fischer , and J. C. Svenning . 2018. “‘Wild’ in the City Context: Do Relative Wild Areas Offer Opportunities for Urban Biodiversity?” Landscape and Urban Planning 170: 256–265. 10.1016/j.landurbplan.2017.09.027.

[ece370704-bib-0061] Naimi, B. 2017. “Package ‘usdm’.” R Package. https://cran.r‐project.org/web/packages/usdm/index.html.

[ece370704-bib-0062] Nazar‐Silva, E. E. , and F. A. B. Silva . 2021. “A Taxonomic Revision of the South American Species of Pseudocanthon Bates, 1887 (Coleoptera: Scarabaeidae: Scarabaeinae: Deltochilini).” Zootaxa 5027, no. 1: 61–86. 10.11646/zootaxa.5027.1.3. 34811245

[ece370704-bib-0063] Nichols, E. , T. A. Gardner , C. A. Peres , S. Spector , and T. S. R. Network . 2009. “Co‐Declining Mammals and Dung Beetles: An Impending Ecological Cascade.” Oikos 118, no. 4: 481–487. 10.1111/j.1600-0706.2009.17268.x.

[ece370704-bib-0064] Nichols, E. , T. Larsen , S. Spector , et al. 2007. “Global Dung Beetle Response to Tropical Forest Modification and Fragmentation: A Quantitative Literature Review and Meta‐Analysis.” Biological Conservation 137: 1–19. 10.1016/j.biocon.2007.01.023.

[ece370704-bib-0065] Nichols, E. , S. Spector , J. Louzada , et al. 2008. “Ecological Functions and Ecosystem Services Provided by Scarabaeinae Dung Beetles.” Biological Conservation 141, no. 6: 1461–1474. 10.1016/j.biocon.2008.04.011.

[ece370704-bib-0066] Noriega, J. A. , C. Zapata‐Prisco , H. García , et al. 2020. “Does Ecotourism Impact Biodiversity? An Assessment Using Dung Beetles (Coleoptera: Scarabaeinae) as Bioindicators in a Tropical Dry Forest Natural Park.” Ecological Indicators 117: 106–580. 10.1016/j.ecolind.2020.106580.

[ece370704-bib-0067] Oksanen, J. , F. G. Blanchet , R. Kindt , et al. 2015. “Vegan: Community Ecology Package.” R Package version 2.3–2.1. 10.4135/9781412971874.n145.

[ece370704-bib-0068] Oliveira‐Filho, A. T. , K. G. Dexter , R. T. Pennington , M. F. Simon , M. L. Bueno , and D. M. Neves . 2021. “On the Floristic Identity of Amazonian Vegetation Types.” Biotropica 53, no. 3: 767–777. 10.1111/btp.12932.

[ece370704-bib-0069] Parris Kirsten, M. 2016. Ecology of Urban Environments. New York, NY: John Wiley & Sons.

[ece370704-bib-0070] Philips, T. K. , E. Pretorius , and C. H. Scholtz . 2004. “A Phylogenetic Analysis of Dung Beetles (Scarabaeinae: Scarabaeidae): Unrolling an Evolutionary History.” Invertebrate Systematics 18, no. 1: 53–88. 10.1071/IS03030.

[ece370704-bib-0071] Podgaiski, L. R. , M. De Souza Mendonça Jr. , and V. De Patta Pillar . 2011. “O Uso de Atributos Funcionais de Invertebrados Terrestres na Ecologia: O Que, Como e Por Quê?” Oecologia Australis 15, no. 4: 835–853. 10.4257/oeco.2011.1504.05.

[ece370704-bib-0072] Püttker, T. , R. Crouzeilles , M. Almeida‐Gomes , et al. 2020. “Indirect Effects of Habitat Loss via Habitat Fragmentation: A Cross‐Taxa Analysis of Forest‐Dependent Species.” Biological Conservation 241: 108368. 10.1016/j.biocon.2019.108368.

[ece370704-bib-0112] QGIS . 2021. “QGIS Geographic Information System.” Open SourceGeospatial Foundation Project. 3.20–1. http://qgis.org.

[ece370704-bib-0073] R Development Core Team . 2022. R: A Language and Environment for Statistical Computing. Vienna, Austria: R Foundation for Statistical Computing.

[ece370704-bib-0074] Ramírez‐Restrepo, L. , and G. Halffter . 2016. “Copro‐Necrophagous Beetles (Coleoptera: Scarabaeinae) in Urban Areas: A Global Review.” Urban Ecosystems 19, no. 3: 1179–1195. 10.1007/s11252-016-0536-2.

[ece370704-bib-0075] Ratcliffe, B. C. 2013. “The Dung‐and Carrion‐Feeding Scarabs (Coleoptera: Scarabaeoidea) of an Amazonian Blackwater Rainforest: Results of a Continuous, 56‐Week, Baited‐Pitfalltrap Study.” Coleopterists Bulletin 67, no. 4: 481–520.

[ece370704-bib-0076] Ribeiro, M. C. , A. C. Martensen , J. P. Metzger , M. Tabarelli , F. Scarano , and M. Fortin . 2011. “The Brazilian Atlantic Forest: A Shrinking Biodiversity Hotspot.” In Biodiversity Hotspots, edited by F. E. Zachos and J. C. Habel , 405–434. Berlin, Heidelberg, Germany: Springer‐Verlag.

[ece370704-bib-0077] Rivera, J. D. , P. G. da Silva , and M. E. Favila . 2021. “Landscape Effects on Taxonomic and Functional Diversity of Dung Beetle Assemblages in a Highly Fragmented Tropical Forest.” Forest Ecology and Management 496: 119390. 10.1016/j.foreco.2021.119390.

[ece370704-bib-0078] Rocca, F. D. , and P. Milanesi . 2022. “The Spread of the Japanese Beetle in a European Human‐Dominated Landscape: High Anthropization Favors Colonization of *Popillia japonica* .” Diversity 14: 658. 10.3390/d14080658.

[ece370704-bib-0080] Salomão, R. P. , F. Alvarado , F. Baena‐Díaz , et al. 2019. “Urbanization Effects on Dung Beetle Assemblages in a Tropical City.” Ecological Indicators 103: 665–675. 10.1016/j.ecolind.2019.04.045.

[ece370704-bib-0081] Salomão, R. P. , A. Arriaga‐Jiménez , and B. Kohlmann . 2021. “The Relationship Between Altitudinal Gradients, Diversity, and Body Size in a Dung Beetle (Coleoptera: Scarabaeinae: Onthophagus) Model System.” Canadian Journal of Zoology 991: 33–43. 10.1139/cjz-2020-0072.

[ece370704-bib-0082] Salomão, R. P. , F. Z. Vaz‐de‐Mello , M. J. Cupello , and L. A. Cassiano . 2024. “Dung Beetle (Coleoptera: Scarabaeinae) Attraction to Woodcreeper (Aves: Dendrocolaptidae) Dropping in the Central Amazon.” Acta Amazonica 54, no. 1: e54bc23151. 10.1590/1809-4392202301510.

[ece370704-bib-0083] Sánchez‐de‐Jesús, H. A. , V. Arroyo‐Rodríguez , E. Andresen , and F. Escobar . 2016. “Forest Loss and Matrix Composition are the Major Drivers Shaping Dung Beetle Assemblages in a Fragmented Rainforest.” Landscape Ecology 314: 843–854. 10.1007/s10980-015-0293-2.

[ece370704-bib-0084] Santana, R. O. , R. C. Delgado , and A. Schiavetti . 2020. “The Past, Present and Future of Vegetation in the Central Atlantic Forest Corridor, Brazil.” Remote Sensing Applications: Society and Environment 20: 100–357. 10.1016/j.rsase.2020.10m.

[ece370704-bib-0085] Santos‐Heredia, C. , E. Andresen , D. A. Zárate , and F. Escobar . 2018. “Dung Beetles and Their Ecological Functions in Three Agroforestry Systems in the Lacandona Rainforest of Mexico.” Biodiversity and Conservation 27: 2379–2394. 10.1007/s10531-018-1542-x.

[ece370704-bib-0086] Sathler, D. , R. L. Monte‐Mór , and J. A. M. Carvalho . 2009. “As Redes Para Além dos Rios: Urbanização e Desequilíbrios na Amazônia Brasileira.” Nova Economia 191: 11–39. 10.1590/S0103-63512009000100002.

[ece370704-bib-0087] Scheffler, P. Y. 2005. “Dung Beetle (Coleoptera: Scarabaeidae) Diversity and Community Structure Across Three Disturbance Regimes in Eastern Amazonia.” Journal of Tropical Ecology 211: 9–19. 10.1017/S0266467404001683.

[ece370704-bib-0088] Scholtz, C. H. , A. L. V. Davis , and U. Kryger . 2009. Evolutionary Biology and Conservation of Dung Beetles. Sofia‐Moscow, Russia: Pensoft.

[ece370704-bib-0089] Seress, G. , and A. Liker . 2015. “Habitat Urbanization and Its Effects on Birds.” Acta Zoologica Academiae Scientiarum Hungaricae 61, no. 4: 373–408. 10.17109/AZH.61.4.373.2015.

[ece370704-bib-0090] Servín‐Pastor, M. , R. P. Salomão , F. Caselín‐Cuevas , et al. 2020. “Malnutrition and Parasitism Shape Ecosystem Services Provided by Dung Beetles.” Ecological Indicators 121: 107205. 10.1016/j.ecolind.2020.107205.

[ece370704-bib-0091] Silva, P. G. , C. A. Nunes , L. F. Ferreira , et al. 2019. “Patch and Landscape Effects on Forest‐Dependent Dung Beetles are Masked by Matrix‐Tolerant Dung Beetles in a Mountaintop Rainforest Archipelago.” Science of the Total Environment 651: 1321–1331. 10.1016/j.scitotenv.2018.09.195. 30360264

[ece370704-bib-0092] Slade, E. M. , D. J. Mann , and O. T. Lewis . 2011. “Biodiversity and Ecosystem Function of Tropical Forest Dung Beetles Under Contrasting Logging Regimes.” Biological Conservation 144: 166–174. 10.1016/j.biocon.2010.08.011.

[ece370704-bib-0093] Souza Jr, C. M. Z. , J. Shimbo , M. R. Rosa , et al. 2020. “Reconstructing Three Decades of Land Use and Land Cover Changes in Brazilian Biomes With Landsat Archive and Earth Engine.” Remote Sensing 1217: 27–35. 10.3390/rs12172735.

[ece370704-bib-0094] Souza, T. B. , F. M. França , J. Barlow , et al. 2020. “The Relative Influence of Different Landscape Attributes on Dung Beetle Communities in the Brazilian Atlantic Forest.” Ecological Indicators 117: 106–534. 10.1016/j.ecolind.2020.106534.

[ece370704-bib-0095] Stavert, J. R. , B. A. Drayton , J. R. Beggs , and A. C. Gaskett . 2014. “The Volatile Organic Compounds of Introduced and Native Dung and Carrion and Their Role in Dung Beetle Foraging Behaviour.” Ecological Entomology 39, no. 5: 556–565. 10.1111/een.12133.

[ece370704-bib-0096] Suárez‐Castro, A. F. , J. S. Simmonds , M. G. Mitchell , M. Maron , and J. R. Rhodes . 2018. “The Scale‐Dependent Role of Biological Traits in Landscape Ecology: A Review.” Current Landscape Ecology Reports 3: 12–22. 10.1007/s40823-018-0031-y.

[ece370704-bib-0097] Tissiani, A. S. D. O. , F. Z. Vaz‐de‐Mello , and J. H. Campelo‐Júnior . 2017. “Dung Beetles of Brazilian Pastures and Key to Genera Identification (Coleoptera: Scarabaeidae).” Pesquisa Agropecuária Brasileira 52: 401–418. 10.1590/S0100-204X2017000600004.

[ece370704-bib-0098] Tocher, M. 1998. “A Comunidade de Anfíbios da Amazônia Central: Diferenças na Composição Específica Entre a Mata Primária e Pastagens.” In Floresta Amazônica: Dinâmica, Regeneração e Manejo, edited by C. Gascon and P. Moutinho , 219–232. Manaus, Brazil: National Institute for Amazonian Research (INPA).

[ece370704-bib-0099] Tolessa, T. , F. Senbeta , and M. Kidane . 2017. “The Impact of Land Use/Land Cover Change on Ecosystem Services in the Central Highlands of Ethiopia.” Ecosystem Services 23: 47–54. 10.1016/j.ecoser.2016.11.010.

[ece370704-bib-0100] Vaz‐de‐Mello, F. V. , G. G. Brown , R. Constantino , et al. 2009. “A Importância da Meso e Macrofauna do Solo na Fertilidade e Como Bioindicadores.” Boletim Informativo da Sociedade Brasileira de Ciência Do Solo 34, no. 1: 39–43.

[ece370704-bib-0101] Vaz‐de‐Mello, F. Z. , W. D. Edmonds , F. C. Ocampo , and P. Schoolmeesters . 2011. “A Multilingual Key to the Genera and Subgenera of the Subfamily Scarabaeinae of the New World (Coleoptera: Scarabaeidae).” Zootaxa 2854: 1–73. 10.11646/zootaxa.2854.1.1.

[ece370704-bib-0102] Venugopal, K. S. , S. K. Thomas , and A. T. Flemming . 2012. “Diversity and Community Structure of Dung Beetles (Coleoptera: Scarabaeinae) Associated With Semi‐Urban Fragmented Agricultural Land in the Malabar Coast in Southern India.” Journal of Threatened Taxa 47: 2685–2692. 10.11609/JoTT.o3074.2685-92.

[ece370704-bib-0103] Viegas, G. , C. Stenert , U. H. Schulz , and L. Maltchik . 2014. “Dung Beetle Communities as Biological Indicators of Riparian Forest Widths in Southern Brazil.” Ecological Indicators 36: 703–710. 10.1016/j.ecolind.2013.09.036.

[ece370704-bib-0104] Vieira, I. C. G. , P. D. Toledo , J. D. Silva , and H. Higuchi . 2008. “Deforestation and Threats to the Biodiversity of Amazonia.” Brazilian Journal of Biology 68: 949–956. 10.1590/S1519-69842008000500004. 19197467

[ece370704-bib-0105] Villaseñor, N. R. , D. A. Dirscoll , M. A. H. Escobar , P. Gibbons , and D. B. Lindenmeyer . 2014. “Urbanization Impacts on Mammals Across Urban‐Forest Edges and a Predictive Model of Edge Effects.” PLoS One 9, no. 5: e97036. 10.1371/journal.pone.0097036. 24810286 PMC4014578

[ece370704-bib-0106] Violle, C. , M. L. Navas , D. Vile , et al. 2007. “Let the Concept of Trait Be Functional!” Oikos 1165: 882–892. 10.1111/j.0030-1299.2007.15559.x.

[ece370704-bib-0107] Vitel, C. S. , P. M. Fearnside , and P. M. L. A. Graça . 2009. “Análise da Inibição do Desmatamento Pelas Áreas Protegidas na Parte Sudoeste do Arco de Desmatamento.” Anais XIV Simpósio Brasileiro de Sensoriamento Remoto, Natal, Brasil, 6377–6384.

[ece370704-bib-0108] Zanette, L. , P. Doyle , and S. M. Trémont . 2000. “Food Shortage in Small Fragments: Evidence From an Area‐Sensitive Passerine.” Ecology 81: 1654–1666. 10.1890/0012-9658(2000)081[1654:FSISFE]2.0.CO;2.

[ece370704-bib-0109] Zungu, M. M. , M. S. Maseko , R. Kalle , T. Ramesh , and C. T. Downs . 2020. “Factors Affecting the Occupancy of Forest Mammals in an Urban‐Forest Mosaic in EThekwini Municipality, Durban, South Africa.” Urban Forestry & Urban Greening 48: 126–562. 10.1016/j.ufug.2019.126562.

